# Radical-induced lipid oxidation produces a torrent of leukotriene-like agonists in severe asthma

**DOI:** 10.1016/j.jaci.2025.09.027

**Published:** 2025-10-15

**Authors:** Si-Yang Liu, Mikhail Linetsky, Abby Hite, Yu-Shiuan Cheng, Masaru Miyagi, Serena C. Zhu, Hong Zeng, Siqi Huang, Myra Qin, Emma Sintic, Carolyn M. Koutures, Abigail Meneses, Olivia R. Laniak, Sailaja Paruchuri, Lakshminarayan R. Teegala, Kaixi Cui, Fariba Rezaee, Robert G. Salomon

**Affiliations:** a the Department of Chemistry, Case Western Reserve University, Cleveland; b the Department of Pharmacology, Case Western Reserve University, Cleveland; c the Department of Physiology and Pharmacology, The University of Toledo, Toledo; d the Department of Inflammation and Immunity, Lerner Research Institute, Cleveland Clinic, Cleveland

**Keywords:** Pseudo leukotriene, cysteinyl leukotriene receptor, biomarker, asthma, inflammation, ERK, Akt, montelukast, zafirlukast

## Abstract

**Background:**

We discovered a family of cysteinyl leukotriene (CysLT)–like biomolecules, pseudo leukotrienes (øLTs), produced through radical-induced oxidation of arachidonate. Objective: We sought to test hypotheses that øLT levels are elevated in asthma and that øLTs induce CysLT receptor–dependent inflammatory signaling in bronchial epithelial cells.

**Methods:**

Liquid chromatography-tandem mass spectrometry analysis determined levels of øLTs in urine from human subjects and in lung samples from mice exposed to house dust mite extract. Inflammatory signaling induced by øLTs and CysLTs in human bronchial epithelial cells were compared.

**Results:**

Mean urinary øLTC and øLTD concentrations were elevated 4-fold (*P* = .004) to 5-fold (*P* = .0015) in severe asthma. Adding øLTC+øLTD improved the statistical significance of the differences between controls and moderate (*P* = .0004) or severe (*P* = .00008) asthma. Pulmonary øLT concentrations were elevated 100% in mice after allergen exposure. øLTs induced phosphorylation of extracellular signal-regulated kinase and protein kinase B (Akt) in bronchial epithelial cells, which was inhibited by CysLT receptor antagonists.

**Conclusions:**

Urinary øLTs are new biomarkers that may facilitate noninvasive assessment of asthma severity and the efficacy of therapeutic measures. Presumably øLTs contribute to CysLT receptor–mediated inflammation through CysLT-like inflammatory biological activities previously ascribed uniquely to CysLTs. øLTs provide a mechanistic link between radical-induced biochemistry, activation of the extracellular signal-regulated kinase/Akt pathway, and asthma. These discoveries require re-examination of long-held disease paradigms for the involvement of CysLTs in asthma pathology and reconsideration of the mechanistic basis for the efficacy of CysLT receptor antagonists for ameliorating chronic asthma. Inhibiting the biosynthesis of øLTs may provide new therapeutic strategies for preventing asthmatic inflammation.

## Capsule summary

The discovery of radical-induced generation of øLTs, new biomarkers of severe asthma with CysLT-like receptor-mediated inflammatory activities, calls for re-examination of long-held disease paradigms for the involvement of CysLTs in asthma pathology.

## GRAPHICAL ABSTRACT

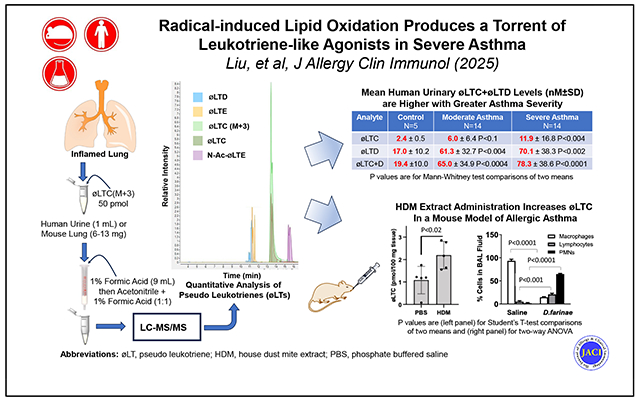

Impaired respiratory tract antioxidant defenses are a hallmark of asthma. Airway glutathione (GSH), an efficient scavenger of lipid free radicals, is depleted in severe asthma.^1^ The antioxidant protein superoxide dismutase (SOD) detoxifies superoxide, which is a reactive oxygen species (ROS) that promotes radical-induced lipid oxidation and fosters damage to DNA, proteins, and lipids. SOD activity is lower in the lungs of patients with asthma than in healthy controls and rapidly decreases during antigen-induced asthmatic response.^2^ Depletion of GSH and active SOD contributes to a vicious cycle of increasing oxidative stress leading to apoptotic loss of airway epithelial cells.^3^ However, the precise mechanisms linking the radical-induced lipid oxidation promoted by ROS to asthma pathogenesis remain poorly understood.

Arachidonate is a target of radical-induced oxidative damage that can cleave its 20-carbon skeleton into reactive fragments. We discovered that adduction of GSH to 1 of these fragments in cells exposed to oxidative stress leads to the generation of a family of cysteinyl leukotriene (CysLT)–like 6-sulfidopeptide derivatives of octanoic acid,^4^ which we refer to as pseudo leukotriene (øLT)C, øLTD, and øLTE because they are structurally analogous to LTC_4_, LTD_4_, and LTE_4_.

We anticipated that high levels of ROS associated with severe asthma would promote lipid oxidation resulting in elevated øLT levels. Furthermore, these biomolecules may exhibit activities similar to the CysLTs, eg, triggering inflammatory signaling in human bronchial epithelial (HBE) cells. We now report that øLT levels in human urine are elevated in severe asthma and are orders of magnitude greater than those of CysLTs. Allergen challenge induces the production of øLTC in the lungs of a murine model of allergic asthma. øLTs exhibit CysLT-like biological activity, inducing inflammatory signaling in HBE cells that is inhibited by the cysteinyl leukotriene receptor (CysLTR) antagonists montelukast or zafirlukast. CysLTR antagonists are widely used to block pathologic activation that contributes to asthma pathobiology.^5^ The present study provides presumptive evidence that blocking CysLTR-mediated inflammation may include øLT-induced pathologic activities previously ascribed uniquely to CysLTs.

## METHODS

### Materials

Hypercarb Porous Graphitic Carbon (1.0 × 100 mm, 3 mm; 35003-101030) equipped with a guard cartridge (10 mm × 2.1, 3 mm; 35003-012101) and Hypercarb Porous Graphitic Carbon (2.1 × 150 mm, 5 mm; 35005-152130) equipped with a guard cartridge (10 mm, 5 mm; 35005-011001) were obtained from Thermo Fisher Scientific (Waltham, Mass). Glass fiber filters (17 mm, 0.45 μm; SF17979) were purchased from Tish Scientific (Cleves, Ohio). Strata-X cartridges (33 μm polymeric reverse phase 30 mg/1-mL tube; 8B-S100-TAK) were procured from Phenomenex (Torrance, Calif). All solvents used in this project for øLT extraction from mouse lung homogenates were of American Chemical Society grade and were purchased from Thermo Fisher Scientific. All solvents used for øLT purification and liquid chromatography (LC) mass spectrometry (MS) analyses were of LC-MS grade and were procured from Thermo Fisher Scientific. House dust mite (HDM) (*Dermatophagoides farinae*) protein extract was purchased from CiteQ biologics, (Groningen, The Netherlands). PBS and Dulbecco modified Eagle medium (10-013-CV) were purchased from Corning (Corning, NY). FBS (26140079) and 1003 antibiotic-antimycotic (15-240-062) were obtained from Gibco (Waltham, Mass). HEPES buffer (SH30237.01) was purchased from Cytiva (Marlborough, Mass). LTD_4_ (20310), LTE_4_ (20410), montelukast (35779), and zafirlukast (10008282) all were purchased from Cayman Chemical (Ann Arbor, Mich). The mammalian cell lysis buffer (GB-180-500), ProBlock protease inhibitor (GB-326-10), and BSA (A-420-1) were obtained from GoldBio (St Louis, Mo). Phosphatase inhibitor cocktails (AAJ63907AA and J61022.AA), bicinchoninic acid protein assay kits (23227), and tris-glycine gels (XP04202BOX) were purchased from Thermo Fisher Scientific. Polyvinylidene difluoride membranes (IPVH00010) were obtained from Sigma-Aldrich (St Louis, Mo). All primary antibodies for Western blotting (detailed in Table E1 in this article’s Online Repository available at www.jacionline.org) were purchased from Cell Signaling Technology (Danvers, Mass), and horseradish peroxidase–conjugated secondary antibodies (111-035-003) were purchased from Jackson ImmunoResearch (West Grove, Pa). The chemiluminescence substrate (1705061) was obtained from Bio-Rad (Hercules, Calif).

### Animals

C57BL/6J mice (Jackson Laboratories, Bar Harbor, Me) were maintained at the Comparative Medicine Unit at Northeast Ohio Medical University. All animal experiments were done in accordance with standard guidelines as approved by the Animal Care and Use Committee of Northeast Ohio Medical University.

### Participants and urine collection

The study population included 5 healthy controls, 15 participants with nonsevere (moderate) asthma, and 15 participants with severe asthma between the age of 18 and 79 years. Participants with asthma were included if they had asthma diagnosed by a medical specialist and/or a history of a positive methacholine test and/or reversibility of >10% of FEV_1_. Asthma was classified as severe or nonsevere based on American Thoracic Society and ERS guidelines.^6,7^ Control participants were included if they had no history of lung disease. Additionally, participants were excluded for diabetes; active smoking status or smoking history of greater than 10 pack-years; or any other significant respiratory disease, cardiac disease, acute or chronic renal failure, or the presence of clinically relevant diseases or comorbidities. All participants provided written informed consent before initiation of study procedures.

Urine samples were collected in the morning, centrifugated at 2000*g*, and aliquoted and stored at −80°C in the Asthma Bio-repository. One set of urinary concentration measurements was normalized by concurrently measured urine creatinine concentrations to account for differences in hydration.

### HDM-induced øLTC production in mouse lung

HDM (*D farinae*) protein extract was resuspended in PBS at a concentration of 2 mg/mL. Following administration of isoflurane, 6- to 8-week-old mice received *D farinae* (25 μg/animal) or saline intranasally, 3 times a week for 5 weeks. Mice were euthanized 24 hours after the last intranasal instillation.

### Extraction of øLTs from human urine

Frozen urine samples were thawed on ice. One milliliter of urine was added to 9 mL of 1% formic acid (FA) in LC-quality water containing N-Ac-øLTE-d_3_ (20 μg/mL; 400 ng/sample) and øLTC M + 3 isotopologue (50 pmol). After filtering the samples through a 17-mm glass fiber filter (0.45 μm; SF17979; Tish Scientific), the mixture was loaded on a preconditioned Strata-X cartridge. A Strata-X cartridge (33 μm polymeric reversed phase 30 mg/1-mL tube; Phenomenex 8B-S100-TAK; Phenomenex) was preconditioned by passing 1 mL of 0.1% FA, followed by 2 mL of 0.1% FA in 50% aqueous solution of acetonitrile and equilibrating the cartridge with 1 mL of 0.1% FA. After sample loading, the cartridge was washed with LC-quality water containing 0.1% FA, and the øLT fraction was eluted with 50% acetonitrile containing 0.1% FA.^4^ After evaporating solvents at room temperature on a SpeedVac (Thermo Fisher Scientific), the samples were reconstituted in 0.1% FA solution (200 μL), vortexed, and sonicated.

### Extraction of creatinine from human urine

To quantify the levels of creatinine in human urine samples, the electrospray ionization (ESI) HPLC-MS-MS method of Hou et al^8^ was used with some modifications. Briefly, frozen urine samples were thawed on ice and vortexed to suspend any settled precipitate. FA (10 μL) was added to a 100-μL aliquot of human urine sample followed by an addition of 890 μL of 0.1% of FA in water, stirred, and centrifuged at 14,000 rpm for 10 minutes at 4°C. The mixture was filtered through a 0.22-μm polyethersulfone membrane filter, and 10 μL of the diluted urine aliquot was transferred to a 5-mL low-binding Eppendorf snap-cap tube. It was spiked with 100 μL of creatinine-D_3_ internal standard solution (4 μg/mL) followed by an addition of 1,890 μL 0.1% of FA in water (1:2,000 final dilution) and vortexing.

### Extraction of øLTs from mouse lung

Lung tissue (6-13 mg) from each mouse was weighed and subsequently homogenized at 0°C in 300 μL of 0.2% aqueous FA containing 0.25 mM of butylated hydroxytoluene and 1 mM of diethylenetriaminepentaacetic acid, and heavy atom labeled synthetic standard glycine-^13^C_2_,^15^N-øLTC (500 fmol in 0.1% FA) was added. The homogenate was extracted at 0°C with 425 μL of dichloromethane/methanol (1:0.75). The aqueous layer was collected, and the organic phase (2 layers) was extracted again with 300 μL of 0.2% aqueous FA containing 0.25 mM of butylated hydroxytoluene and 1 mM of diethylenetriaminepentaacetic acid at 0°C. The combined aqueous extracts were dried on a SpeedVac at room temperature and reconstituted in 1 mL of 0.1% FA. The acidified aqueous extract was then loaded onto the solid phase extraction column: Strata X-C column packed with of Strata X- polymeric C18 material (30 mg/1-mL cartridge; Phenomenex) as described above.^4^ The last eluate from the Strata-X column (bound fraction) was concentrated to ≈10 μL by SpeedVac and then reconstituted to 50 μL with 0.1% FA in water before LC-MS/MS injection.

### Cell culture and treatment with øLTs or LTs in the presence or absence of inhibitors

The HBE cell line 16HBE14o- (16HBE) was cultured in Dulbecco modified Eagle medium supplemented with 10% FBS, 0.5% antibiotic-antimycotic solution, and 25 mM HEPES buffer. Cells were maintained in a humidified incubator at 37°C under 5% CO_2_, and the culture medium was replaced every 48 hours. On reaching 75% to 80% confluence, cells were treated with either synthetic øLTs prepared in PBS or CysLTs dissolved in absolute ethanol for comparative analyses. For inhibition studies, cells were preincubated with 1 μM of the CysLT receptor antagonists montelukast or zafirlukast dissolved in dimethyl sulfoxide for 1 hour before øLTs treatment. Vehicle-only controls were included to account for potential solvent effects. All experiments were performed in quadruplicate.

### LC-MS/MS analysis of creatinine levels in human urine

Urinary CysLT levels are generally reported relative to those of creatinine. The Jaffe method for serum creatinine measurement is much more practical and cheaper than LC-MS/MS, and therefore it was used for routine practice. However, we used tandem mass spectrometric detection of creatinine, which is a more accurate and specific method that is used for verification of discordant clinical results and to avoid possible interferences.^9^ An aliquot (containing 2 ng of the internal standard per injection) of the above extract was injected on-column for LC-MS/MS detection. A Hypercarb 2.1 × 150 mm column was used for the analysis with an H_2_O/acetonitrile gradient at the flow rate of 0.3 mL/min as shown in Table E2 (in the Online Repository at www.jacionline.org). Three aliquots were injected for each sample, and the average value of 3 runs was used for calculations. Retention times were 8.195 to 8.205 minutes for creatinine and 8.09 to 8.195 minutes for creatinine-D_3_ (see Fig E5, A, in this article’s Online Repository at www.jacionline.org). For detection of creatinine, ESI MS was performed on an Agilent 6460 Triple Quad mass spectrometer (Agilent Technologies, Santa Clara, Calif) coupled with an Agilent 1290 Series UHPLC G4220A Binary Pump (Agilent Technologies) operated in a positive ion mode with multiple reaction monitoring (MRM) using nitrogen as the sheath and auxiliary gas. The heated capillary temperature was 300°C, the capillary voltage was 2000 V^+^/3500 V^−^. All data were processed with the Agilent MassHunter software (Agilent Technologies). Optimized parameters for detecting each analyte were determined using authentic samples. MS scan time was set at 100 ms for 1 cycle. The optimum collision energy and other parameters were determined for each individual analyte. MS/MS experiments were performed by selecting an ion with an isolation width of 2 m/z. Creatinine and creatinine-D_3_ were detected by selected reaction monitoring using the following transitions: creatinine, m/z 114.1 → 44.2; creatinine-D_3_, m/z 117.1 → 47.2 (internal standard) (Table E3 in the Online Repository at www.jacionline.org).

### LC-MS/MS analysis of øLT levels in human urine

ELISA overestimates CysLT levels in biological extracts. Previously, putative mean levels of CysLTs detected in supernatants from human alveolar monocyte-derived macrophages exposed to HDM extract were 43-fold higher measured by ELISA than by LC-MS/MS (5.6 ng/mL vs 0.13 ng/mL).^10^ Perhaps antibody cross-reactivity, eg, with øLTs, which can be orders of magnitude more abundant than LTs, contributes to this pitfall. Therefore, the quantitation of øLTs in human urine was performed in a manner similar to the one described above for creatinine using the same LC-MS/MS setup. After drying the samples at room temperature on a SpeedVac, they were reconstituted in 0.1% FA solution (200 μL), vortexed, and sonicated, and one-tenth of a sample was injected onto the Hypercarb column (Table E2) for LC-MS/MS detection. Each sample was injected 3 times, and the average value of 3 runs was used for calculations. ESI MS for detection of øLTs was performed on an Agilent 6460 Triple Quad mass spectrometer (Agilent Technologies) coupled to an Agilent 1290 Series UHPLC G4220A Binary Pump (Agilent Technologies) operated in either a positive or a negative ion mode with MRM. The optimum collision energy and other parameters were determined for each individual analyte (Table E3). MS/MS experiments were performed by selecting an ion with an isolation width of 2 m/z. øLTs were detected by selected reaction monitoring using the following transitions: øLTC, m/z 482.2 → 335.1; øLTC-(glycine-^13^C_2_,^15^N, M + 3), m/z 485.2 → 338.1 (internal standard, see Fig E6 in this article’s Online Repository at www.jacionline.org); øLTD, m/z 335.1 → 232.1; øLTE, m/z 296.2 → 278.1; N-acetyl-øLTE, m/z 336.1 → 207.1; and N-d^3^-acetyl-øLTE, m/z 339.1 → 81.1 (internal standard) in the negative ion mode (see Table E3 and Fig E5, B).

### LC-MS/MS analysis of øLTC levels in mouse lung

øLTC quantitation was achieved by using a Hypercarb 1 × 100 mm column flowing H_2_O/acetonitrile gradient at the flow rate of 0.06 mL/min as shown in Table E4 (in the Online Repository at www.jacionline.org). ESI MS for detection of øLTC was performed on an Agilent 6460 Triple Quad mass spectrometer (Agilent Technologies) coupled to an Agilent 1290 Series UHPLC G4220A Binary Pump (Agilent Technologies) operated in the positive ion mode with MRM as described for human urine samples (see above). øLTC was detected by selected reaction monitoring using the following transitions: øLTC, m/z 482.2 → 335.1, and øLTC-(glycine-^13^C_2_,^15^N, M + 3), m/z 485.2 → 338.1 (internal standard), respectively (Table E3).

### Western blot analysis of cell lysates

Total cell lysates were prepared using mammalian cell lysis buffer supplemented with protease inhibitor and phosphatase inhibitor cocktails. Protein concentrations were determined by using bicinchoninic acid assay. Equal amounts of protein (15 μg per lane) were loaded to 4% to 20% tris-glycine gradient and transferred onto polyvinylidene difluoride membranes. Membranes were blocked with 5% BSA and prepared in Trisbuffered saline with 0.1% Tween-20 (TBST) for 1 hour at room temperature, followed by overnight incubation at 4°C with primary antibodies. After five 5-minute washes with TBST, membranes were incubated with horseradish peroxidase–conjugated secondary antibodies for 1 hour at room temperature. Following additional washes with TBST, signals were developed using enhanced chemiluminescence substrate and visualized using a Li-Cor Odyssey Fc imaging system (Li-Cor, Lincoln, Neb). Quantitative densitometric analysis of the protein bands was performed using Image Studio software (Li-Cor).

### Statistical analysis

For determination of urinary and mouse lung øLTs, the LC-MS/MS analyses were performed in triplicate. For determination of phosphorylation levels by Western blots, all experiments were performed in quadruplicate. Kolmogorov-Smirnov normality test was applied to the data for øLT levels in urine. Because non-normal distributions were found (Data E3 in the Online Repository at www.jacionline.org), significant differences were determined by Mann-Whitney nonparametric analysis. For changes in protein phosphorylation levels, statistical significance of pairwise differences between cohorts was determined by 1-way ANOVA. *P* value < .05 was considered statistically significant. Data are presented as mean ± SD.

## RESULTS

### Human urinary levels (pmol/mg creatinine) of øLTC and øLTD are elevated in severe asthma

**FIG 1.**
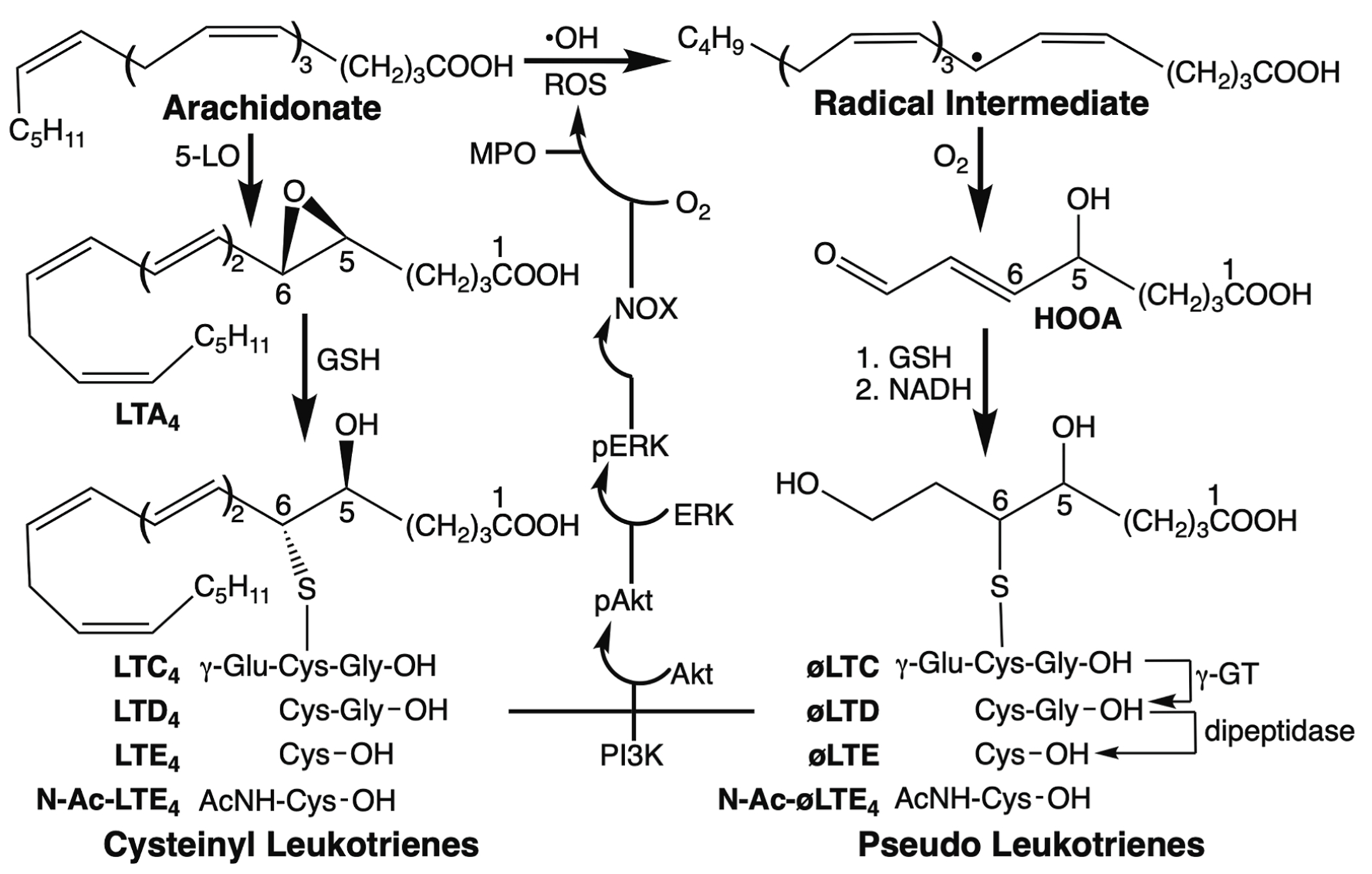
Biosynthesis and pro-oxidant signaling of cysLTs and øLTs. ROS initiate øLT formation. *NADH,* nicotinamide adenine dinucleotide; *pAkt,* phosphorylated Akt; *pERK,* phosphorylated ERK; *PI3K,* phosphatidylinositol 3-kinase.

**FIG 2.**
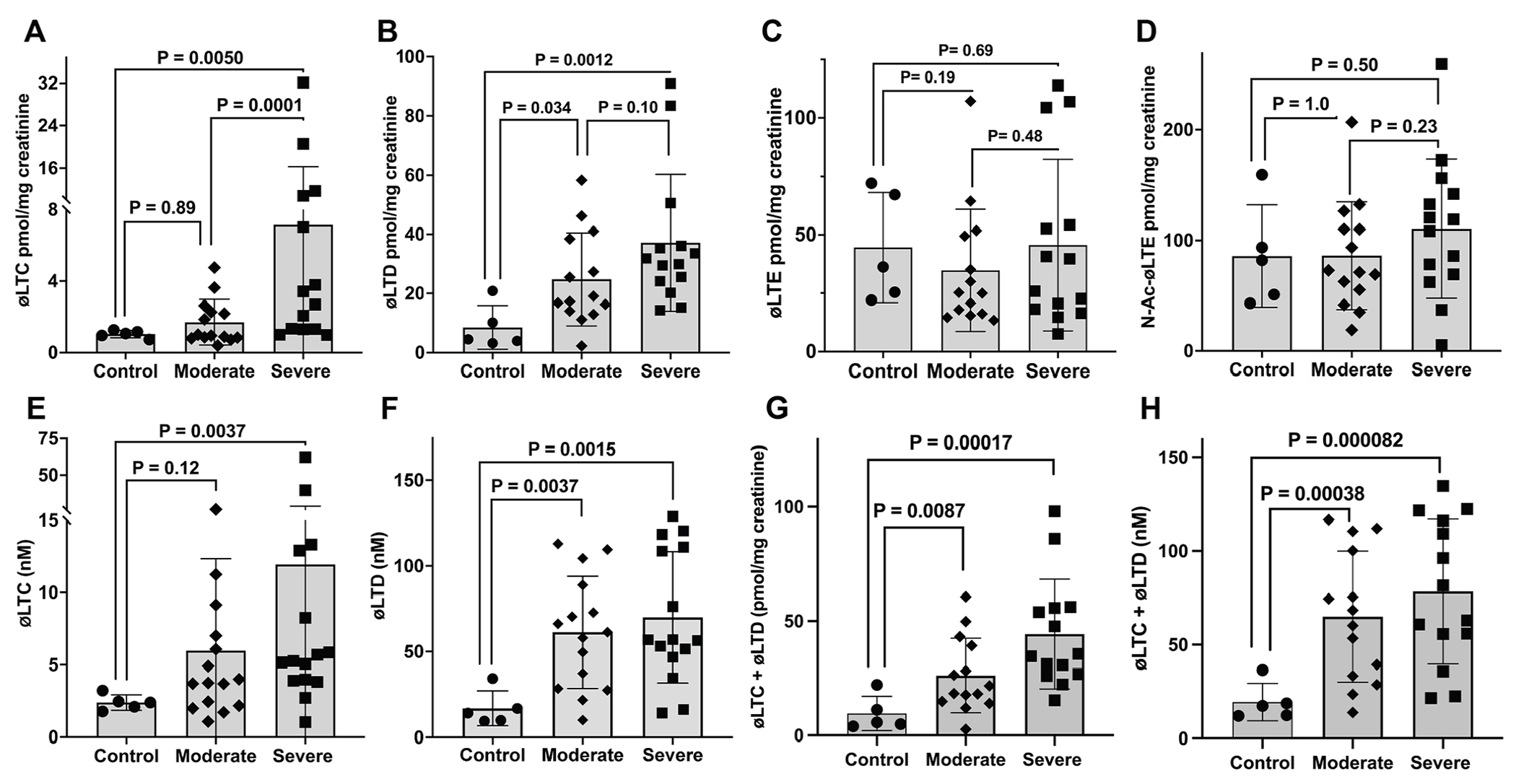
øLT levels are elevated in urine from asthma patients. **(A)** øLTC. **(B)** øLTD. **(C)** øLTE. **(D)** N-Ac-øLTE (pmol/mg creatinine). **(E)** øLTC (nM). **(F)** øLTD (nM). **(G)** øLTC+øLTD (pmol/mg creatinine). **(H)** øLTC+øLTD (nM). Each data point is the mean from 3 independent analyses of each sample, and standard deviations are of those means (see Data E1). Because Kolmogorov-Smirnov normality test showed non-normal distributions (Data E3), significant differences were determined by Mann-Whitney nonparametric analysis. *P* value < .05 is considered statistically significant. Control n = 5, moderate n = 14, and severe n = 14. Data are presented as mean ± SD.

We determined urinary concentrations of øLT and creatinine by LC-MS/MS using the isotope dilution technique with ^13^C_2_,^14^N-la-beled øLTC, N-(^2^H_3_)-acetyl-LTE, and creatinine-D_3_ as the internal standards. Mean urinary øLT levels (pmol/mg creatinine) in severe asthma increased from the putative initial biosynthetic product øLTC (Fig 1) to øLTD to øLTE and to the ultimate metabolite N-acetyl-øLTE: øLTC = 7.1, øLTD = 37, øLTE = 46, N-acetyl-øLTE = 111 (Fig 2, A–D). Mean urinary levels (pmol/mg creatinine) of øLTC were increased 4.2-fold (*P* = .036) in severe compared with nonsevere (moderate)^6,7^ disease (Fig 2, A). Mean levels of both øLTC and øLTD exhibited significant increases, 7.1-fold (*P* = .027) and 4.6-fold (*P* = .016), respectively, for patients with severe asthma versus controls (Fig 2, A and B). These results are summarized in Table I together with data on fractional exhaled nitric oxide (ppb) and lung function (FEV_1_/forced vital capacity [FVC] ratio; %) for each cohort. Nonparametric Spearman correlation tests revealed a weak positive correlation between øLTC and fractional exhaled nitric oxide (*r* = 0.19) and between øLTC+øLTD (nM) and percentage of blood eosinophils (*r* = 0.11) (Data E4 in the Online Repository at www.jacionline.org).

### Urinary concentrations (nM) of øLTC+øLTD are strongly correlated with asthma severity

Nonparametric Spearman correlation test for øLTC versus øLTD (nM) found a moderate positive correlation (*r* = 0.44) that was significant (*P* = .0076). This is consistent with the view that both markers will be elevated in proportion to disease severity. In contrast, nonparametric Spearman correlation test for øLTC versus øLTD (pmol/mg creatinine) found a weak positive correlation (*r* = 0.11) that was not significant (*P* = .53). This is consistent with the view that øLT levels scaled by creatinine concentrations (pmol/mg creatinine) are less reliable markers of disease severity than øLT concentrations (nM).

Levels of urinary biomarkers are usually reported relative to creatinine. However, common therapeutic measures for asthma can impact creatinine concentrations. Patients treated with a combination of glucocorticoids and beta-agonists, eg, albuterol, exhibit 40% higher (*P* = .001) mean urinary creatinine compared with untreated controls.^11^ A nonparametric Spearman correlation test revealed that creatinine concentration (mg/mL) was weakly positively correlated with øLTC (nM) (*r* = 0.36) and moderately positively correlated with øLTD (nM) (*r* = 0.46) and with øLTC+øLTD (nM) (*r* = 0.43). Consequently, dividing øLT concentrations, which increase with asthma severity, by creatinine concentrations, which increase with bronchodilator exposure, can diminish the differences between urinary levels in healthy controls and patients with severe asthma. Regarding øLT levels (pmol/mg creatinine), mean urinary øLT concentrations (nM) increased with asthma severity: øLTC = 12 (Fig 2, E), øLTD = 70 (Fig 2, F), øLTE = 100, and N-acetyl-øLTE = 190 (see Fig E1 in this article’s Online Repository at www.jacionline.org). In contrast, a previous study established that neither urinary LTC_4_ nor urinary LTD_4_ levels were significantly elevated in urine from patients with asthma compared with controls.^12^

Because øLTC is rapidly (half-life = 90 minutes [data not shown]) converted to øLTD in human plasma at 37°C, urinary øLTC levels can be decreased and urinary øLTD levels increased to various extents. We considered that the sum of øLTC+øLTD might provide a superior gauge of øLT production. We found that the *P* values for differences between the mean levels (pmol/mg creatinine) of øLTC+øLTD (Fig 2, G) were an order of magnitude greater than those for øLTC or øLTD alone. Most notably, the *P* values for differences between the mean concentrations (nM) of øLTC+øLTD (Fig 2, H) were another order of magnitude greater than those between the mean levels (pmol/mg creatinine). Of patients with severe asthma, 80% exhibited urinary øLTC+øLTD concentrations that were higher than the maximum found for controls.

### Study population exhibits classic measures of lung function and inflammation

Our patient cohorts exhibited the expected classic measures of impaired lung function and inflammation. Analysis of lung function as FEV_1_/FVC (%) showed lower FEV_1_/FVC (%) corresponding to impaired lung function for patients with severe asthma compared with controls (*P* = .032) (Fig E7 in the Online Repository at www.jacionline.org). For our study subjects, a nonparametric Spearman correlation test revealed a negative correlation (*r* = −0.23), albeit weak, between impaired lung function as indicated by low FEV_1_/FVC (%) and levels of exhaled nitric oxide, a marker of inflammation. As expected, decreased lung function was associated with increased inflammation that, in turn, promoted free radical–induced lipid oxidation leading to øLT biosynthesis.

### Allergen exposure elevates pulmonary inflammation and øLT levels in mice

Mice were sensitized by intranasal treatment with HDM extract in PBS followed by repeated challenge with HDM extract to induce pulmonary inflammation as described previously.^13^ Control mice were treated with PBS alone. Intranasal inhalation of *D farina* antigen resulted in the infiltration of immune cells to the lung and increased PMNs (eosinophils and neutrophils) and lymphocytes in bronchoalveolar lavage fluid as determined by total and differential cell counts (Fig E8 in the Online Repository at www.jacionline.org).^13^ Further, HDM-treated mice exhibited enhanced airway wall thickening as well as collagen deposition and increased expression of IL-13, IL-5, and TNF-α transcripts in their lung tissues.^13^ Allergen exposure induced a 100% increase (*P* = .0201) in mean øLTC levels in mouse lung (see Fig E2 and Data E2 in this article’s Online Repository at www.jacionline.org). Mean øLTC levels in allergen-exposed mice were 2.2 pmol/100 mg of lung tissue, approximately 20 nM.

### øLTs induce inflammatory signaling in HBE cells

Airway epithelial cells are important for orchestrating inflammatory responses in the allergic inflamed lung.^14^ Identifying the endogenous molecules that induce inflammatory activities in these cells remains a challenge. ROS upregulate phosphorylation of nuclear factor-κB (NF-κB),^15,16^ protein kinase B (also known as Akt), and extracellular signal-regulated kinase (ERK) that can be blocked by an inhibitor of ROS generation.^17^ Because ROS initiate the biosynthesis of øLTs, we considered the possibility that øLTs are a missing link between ROS and activation of ERK, Akt, and NF-κB. The first indication that øLTs can induce Cys-LT receptor mediated activation of ERK, Akt, and NF-κB was observed in retinal pigment epithelial cells.^18^ Furthermore, exposure of the human bronchial epithelial cell line 16HBE to LTD_4_ increases phosphorylation of ERK.^19^ Because of its structural analogy to LTD_4_, it seemed plausible that øLTD would have similar biological activity.

**FIG 3.**
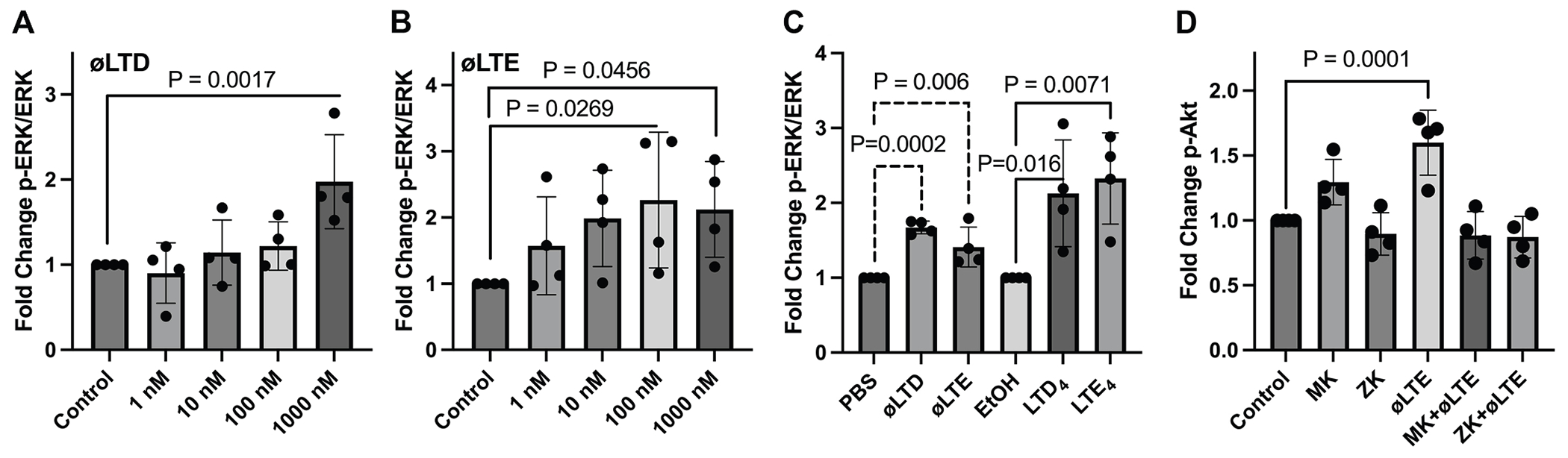
Both LTs and øLTs induce signaling in 16HBE cells that is inhibited by CysLTR antagonists. **(A** and **B)** Concentration dependence of ERK phosphorylation induced by 10-minute exposure to *(A)* øLTD or *(B)* øLTE. **(C)** ERK phosphorylation induced by 200 nM øLTD or øLTE or 100 nM LTD_4_ or LTE_4_. **(D)** Akt phosphorylation induced by øLTE (200 nM) with or without inhibition by pretreatment (1 hour) with montelukast (1 μM) or zafirlukast (1 μM). Data are mean ± SD from 4 independent experiments. Statistical significance of pairwise comparisons calculated by 1-way ANOVA. For representative immunoblotting images, see Fig E4 (in the Online Repository at www.jacionline.org). *EtOH,* Ethyl alcohol; *MK,* montelukast; *pERK,* phosphorylated ERK; *ZK,* zafirlukast.

**FIG 4.**
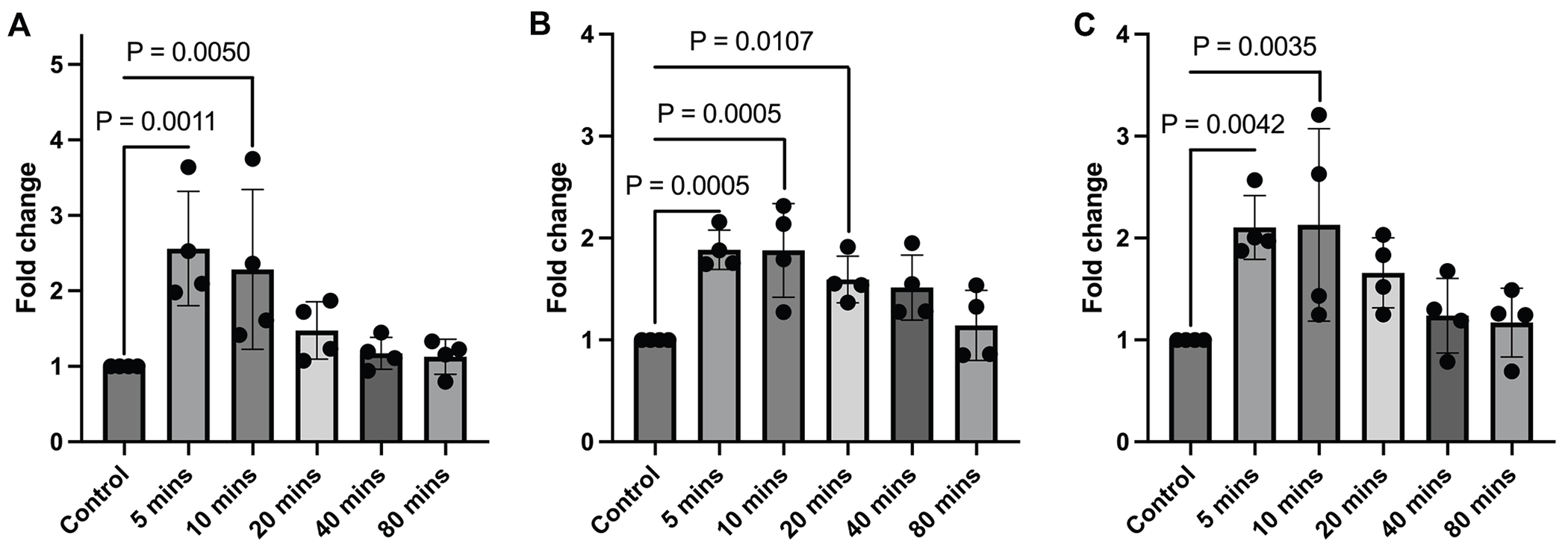
øLT-induced phosphorylation of ERK, Akt, and NF-κB in 16HBE cells exposed to 200 nM øLTD. **(A)** ERK phosphorylation. **(B)** Akt phosphorylation. **(C)** NF-κB phosphorylation. Data are mean ± SD of 4 independent experiments. Statistical significance of pairwise comparisons calculated by 1-way ANOVA. For representative immunoblotting images, see Fig E4 (in the Online Repository at www.jacionline.org).

Although exposure to 100 nM CysLTs suffices to elicit cellular responses in 16HBE cells,^19^ a dose-response study indicated that 100 nM øLTD does not significantly elevate phosphorylated ERK/ERK (Fig 3, A). However, exposure to 200 nM øLTD induced a significant (*P* = .0002) increase in phosphorylated ERK/ERK (Fig 3, C). Thus, 200 nM øLTD is only slightly less effective than 100 nM LTD_4_ (1.7-fold vs 2.1-fold) (Fig 3, C). Similarly, 200 nM øLTE induces significant increases in phosphorylation of ERK (*P* = .006) (Figs 3, B and C) and Akt (*P* = .0001) (Fig 3, D) in 16HBE cells. Both CysLTR antagonists montelukast and zafirlukast abolished the øLTE-induced upregulation of pAkt (Fig 3, D). Upregulation of ERK, Akt, and NF-κB phosphorylation induced by exposure of 16HBE cells to 200 nM øLTD was rapid, reaching a maximum after 5 to 10 minutes (Fig 4). In a murine model of allergic airway disease, NF-κB activation occurred rapidly predominantly in the epithelial cells of the conducting airways. Evidence from clinical studies support the significance of NF-κB activation in asthma.^20,21^ Similar responses were elicited by exposure of 16HBE cells to 200 nM øLTE (Fig E3 in the Online Repository at www.jacionline.org).

## DISCUSSION

Our studies demonstrated that LC-MS/MS analysis readily allows quantitation of øLTs in human urine and mouse lung and that øLTs mimic the ability of CysLTs to induce CysLTR-dependent cellular signaling in 16HBE cells. The significance of øLTs compared with CysLTs for asthma pathogenesis depends on their relative abundances and abilities to induce CysLTR signaling.

### Relative abundance of øLTs and CysLTs

A cohort of patients with steroid-resistant asthma exhibited the highest mean urinary CysLT levels in a previous clinical study.^12^ Mean levels of LTC_4_, LTD_4_, and LTE_4_ corresponded to 0.028 pmol/mg, 0.027 pmol/mg, and 0.081 pmol/mg of creatinine, respectively. These CysLT levels are more than 2 orders of magnitude lower than the mean urinary øLT levels found in the present clinical study (Table I). This suggests, but remains to be confirmed, that urinary øLT levels in individual patients are orders of magnitude higher than CysLT levels. Disease-related increases in mean øLTC and øLTD levels (7.1- and 4.6-fold) are highly significant (*P* = .0050 and *P* = .0012, respectively). Thus, these metabolites are new biomarkers that may facilitate noninvasive assessment of asthma severity and the efficacy of therapeutic measures.

### Comparison of the biological activities of øLTs and CysLTs

The apparent slightly lower biological activities of øLTs compared with CysLTs (Fig 3, A) may indicate that 3 of the 4 øLT diastereomers that do not possess the same absolute configurations as CysLTs are less active than the 1 øLT diastereomer that does. Although the ability of øLTs to induce inflammatory signaling may be an order of magnitude lower than that of CysLTs because their levels in severe asthma are 2 or more orders of magnitude greater than those of CysLTs, the production and biological activities of øLTs may be the dominant factor impacting pathologic CysLTR-dependent inflammation. Although the therapeutic utility of CysLTR antagonists is unaffected by our discoveries, new therapeutic approaches targeting the biosynthesis of øLTs deserve attention.

### Synergism of ROS-dependent øLT biosynthesis and CysLTR-dependent ROS production

#### CysLT biosynthesis.

CysLTs were originally referred to as “slow reactive substance in anaphylaxis”^22^ and subsequently called leukotrienes because they are generated by leukocytes and exhibit intense UV light absorption indicative of a conjugated triene that enabled their initial detection.^23^ Establishing their molecular structures remained elusive for decades. That they are a family of 6-sulfidopeptide derivatives of arachidonate, LTC_4_, LTD_4_, and LTE_4_ (Fig 1), followed the demonstration that GSH is incorporated in their biosynthesis^24^ and mass spectroscopic comparison with standards, provided by unambiguous chemical syntheses.^25–27^ The biosynthesis of CysLTs is not a free radical–induced process that is initiated by ROS. Rather, it is initiated by a 5-lipoxygenase-catalyzed oxidation that generates an epoxide intermediate, LTA_4_ (Fig 1). Adduction of GSH to LTA_4_ produces LTC_4_. γ-Glutamyl transpeptidase–catalyzed isopeptidolysis of LTC_4_ delivers LTD_4_. A dipeptidase catalyzes conversion of LTD_4_ into LTE_4_ that is N-acetylated to deliver N-Ac-LTE_4_.

#### ROS-dependent øLT biosynthesis.

The lack of readily detectable UV absorption and their biological activities that mimic those of CysLTs may account for the fact that øLTs have gone unnoticed until recently. The biosynthesis of øLTs begins with radical-induced oxidative cleavage of arachidonate such as that initiated by ROS (Fig 1). The ensuing adduction of GSH results in a mixture of 4 diastereomeric øLTCs, of which only 1 has the same absolute configurations as LTC_4_.^4^ øLTD is presumably generated by γ-glutamyl transpeptidase–catalyzed isopeptidolysis of øLTC, dipeptidase-catalyzed peptidolysis of øLTD, and N-acetylation of øLTE in analogy with the catabolism of LTC_4_.

#### CysLTR-dependent ROS biosynthesis.

Activation of HBE, airway smooth muscle, and mast cells contributes to the asthmatic inflammatory environment that is characterized by elevated ROS levels. ROS are produced enzymatically by airway epithelial cells^28^ and by phagocytes that have been recruited to the site of inflammation.^29^ The CysLTR1 agonist LTD_4_ upregulates IgE-mediated Akt and ERK phosphorylation in mast cells in the asthmatic lung.^30^ CysLTs can promote ROS production in mast cells by activating nicotinamide adenine dinucleotide oxidase (NOX) through the phosphatidylinositol 3-kinase, Akt, and ERK pathway,^31,32^ which is also triggered by CysLTs in HBE and airway smooth muscle cells.^17,33,34^ The discovery that øLTs mimic the ability of CysLTs to induce Akt/ERK signaling allows øLTs to participate in a vicious cycle of ROS-induced øLT production and øLT-induced activation of the phosphatidylinositol 3-kinase, Akt, and ERK pathway resulting in NOX-dependent ROS generation in the asthmatic inflammatory environment (Fig 1). Exposure of HBE cells to HDM causes mitochondrial dysfunction resulting in increased mitochondrial ROS and the induction of ferroptosis,^35^ which involves accumulation of free intra-cellular iron. This leads to excessive iron-dependent lipid peroxidation and ultimately cell death.^36^ Stimulation of ROS generation by HDM in airway epithelial cells can provide the spark that ignites the cycle of øLT generation through ROS radical–induced lipid oxidation and øLT-induced ROS production. Because radical-induced lipid oxidation is a chain reaction, and propagation steps can generate more than 1 chain carrying radicals, radical-induced lipid oxidation can drive a vicious cycle of ever-increasing inflammation and disease progression.

### Potential therapeutic strategies for preventing øLT biosynthesis

Based on the hypothesis that øLTs can be dominant CysLT receptor agonists, inhibiting radical-induced biosynthesis of øLTs is expected to decrease øLT levels and the associated inflammatory pathology. Hydrogen atom transfer from GSH to the hydroxyl radical is extremely fast, virtually at the diffusion-controlled rate limit.^37^ Therefore, GSH can neutralize ROS, eg, hydroxyl radical, precluding their reaction with arachidonate to generate the radical intermediate in øLT biosynthesis (Fig 1). In addition to its well-recognized activity as a beta-lactamase inhibitor, cilastatin is a potent inhibitor of dipeptidase-mediated depletion of GSH.^38^ A therapeutic intervention that included levofloxacin in combination with imipenem and cilastatin was successfully used in the treatment of a patient with severe persistent asthma and *Legionella* pneumonia.^39^ In retrospect, it is possible that the beneficial effects of cilastatin included the reversal of GSH depletion and the consequent decrease in levels of inflammatory øLTs.

Lung fibrosis usually occurs in the pathogenesis of fatal and long-term asthma and is associated with disease severity and resistance to therapy.^40^ In a murine model of lung fibrosis, bleomycin stimulation induced oxidative stress including decreased lung tissue SOD activity, increased NOX activity, and elevated lipid oxidation.^41^ Paroxetine, a potent inhibitor of myeloperoxidase,^42^ significantly decreased collagen deposition, inflammation, and oxidative stress in lung tissues from mice with bleomycin-induced pulmonary fibrosis.^41^ Myeloperoxidase (MPO) (Fig 1) induces production of ROS that initiate generation of the radical intermediate in øLT biosynthesis. In retrospect, it is possible that the beneficial effects of paroxetine include prevention of MPO-induced ROS production and the consequent initiation of øLT biosynthesis.

### Limitations

The significance of øLTs compared with CysLTs for asthma pathogenesis depends on their relative abundances and abilities to induce CysLTR signaling. Whereas a previous study observed that neither urinary LTC_4_ nor urinary LTD_4_ levels are significantly elevated in urine from patients with asthma compared with controls,^12^ the present study found that urinary levels of øLTs are orders of magnitude greater than those of CysLTs found in that study and are correlated with disease severity. However, an unambiguous conclusion requires measurement of both øLTs and the corresponding CysLTs in urine from the same individuals. The abilities of øLTs and the corresponding CysLTs to induce CysLTR signaling may also differ between cell types and between various CysLT receptors.

The present study did not characterize PMNs as eosinophils or neutrophils or their relative abundance. Future studies must compare the proclivities toward øLT generation in neutrophilic versus eosinophilic asthma. An HDM/LPS-induced steroid-resistant mouse model of neutrophilic airway inflammation is expected to favor øLT production because MPO activity is elevated 3.5-fold (*P* > .01) in this model,^43^ and our previous research demonstrated that MPO promotes the oxidative cleavage of arachidonate to 5-hydroxy-8-oxo-6-octenoic acid,^44^ the precursor of øLTs (Fig 1).

### Conclusions

The present study establishes that the production and CysLT-like biological activities of øLTs are a previously unknown mechanistic link between radical-induced biochemistry and asthma. øLTs are present in human urine. If it is confirmed that urinary øLT levels in individual patients are orders of magnitude higher than CysLT levels, the possibility must be explored that øLTs are the dominant factor impacting pathologic CysLTR-dependent inflammation in severe asthma.

Long-held disease paradigms for the involvement of CysLTs in asthma pathology must be reexamined. øLTs were “unknown unknowns,” confounding factors, that require reconsideration of the mechanistic basis for the efficacy of CysLTR antagonists in ameliorating asthma pathology. Our discoveries that øLTs are upregulated in asthma and that øLTs induce CysLTR-dependent cell signaling can inspire new avenues of inquiry leading to improved outcomes for patients with asthma. The inflammatory asthmatic environment favors the radical biochemistry that produces øLTs. Besides elevated ROS and depleted antioxidant proteins, depletion of airway GSH, a potent radical scavenger, is associated with severe asthma.^1^ Monitoring urinary øLT levels may be useful for identifying nascent disease, especially in genetically susceptible individuals; provide a measure of therapeutic efficacy; and facilitate preventive correction of defective antioxidant defenses before the disease progresses.

## Supplementary Material

1

## Figures and Tables

**TABLE I.** Mean human urinary øLT levels and severe asthma versus control ratios

Category	Mean urinary øLT levels (pmol/mg creatinine) and covariates				
	øLTC	øLTD	øLTE	Feno (ppb)	FEV_1_/FCV ratio (%)
Severe	7.1	37	46	44	77.1
Moderate	1.7	25	35	41	82.8
Control	1.0	8.0	45	21	94.7
Severe/control	7.1	4.6	1.0	2.1	—

Note. øLT levels are from data in Fig 2.

*Feno,* Fractional exhaled nitric oxide.

## Data Availability

Aliquots of the urine samples analyzed in these studies are stored in the senior author’s (R.G.S.) laboratory. They were provided under a Material Transfer Agreement between Case Western Reserve University and the Cleveland Clinic Foundation. Their further distribution would require an Material Transfer Agreement with Cleveland Clinic Foundation. All data are available in the main text or the Online Repository (at www.jacionline.org).

## Clinical implications:

øLTs are biomarkers of asthma severity with potential utility for assessing the efficacy of therapeutic interventions that inhibit their radical-induced biosynthesis and their induction of CysLT receptor–dependent inflammatory signaling.

## Abbreviations used

CysLT: Cysteinyl leukotriene
CysLTR: Cysteinyl leukotriene receptor
ERK: Extracellular signal-regulated kinase
ESI: Electrospray ionization
FA: Formic acid
FVC: Forced vital capacity
GSH: Glutathione
HBE: Human bronchial epithelial
HDM: House dust mite
LC: Liquid chromatography
MPO: Myeloperoxidase
MRM: Multiple reaction monitoring
MS: Mass spectrometry
NF-κB: Nuclear factor-κB
NOX: NADPH oxidase
øLT: Pseudo leukotriene
ROS: Reactive oxygen species
SOD: Superoxide dismutase
TBST: Tris-buffered saline with 0.1% Tween-20
