# Supplementary material for: Radical-induced lipid oxidation produces a torrent of leukotriene-like agonists in severe asthma
Source: J Allergy Clin Immunol. Author manuscript; Available in PMC 2026 Aug 6. (PMC13446106; doi:10.1016/j.jaci.2025.09.027)
Supplement: Supplementary file 1 [file NIHMS2184670-supplement-1.pdf]

Radical-induced Lipid Oxidation Produces a Torrent of Leukotriene-like Agonists in Severe Asthma

SUPPLEMENTARY MATERIALS

Figures

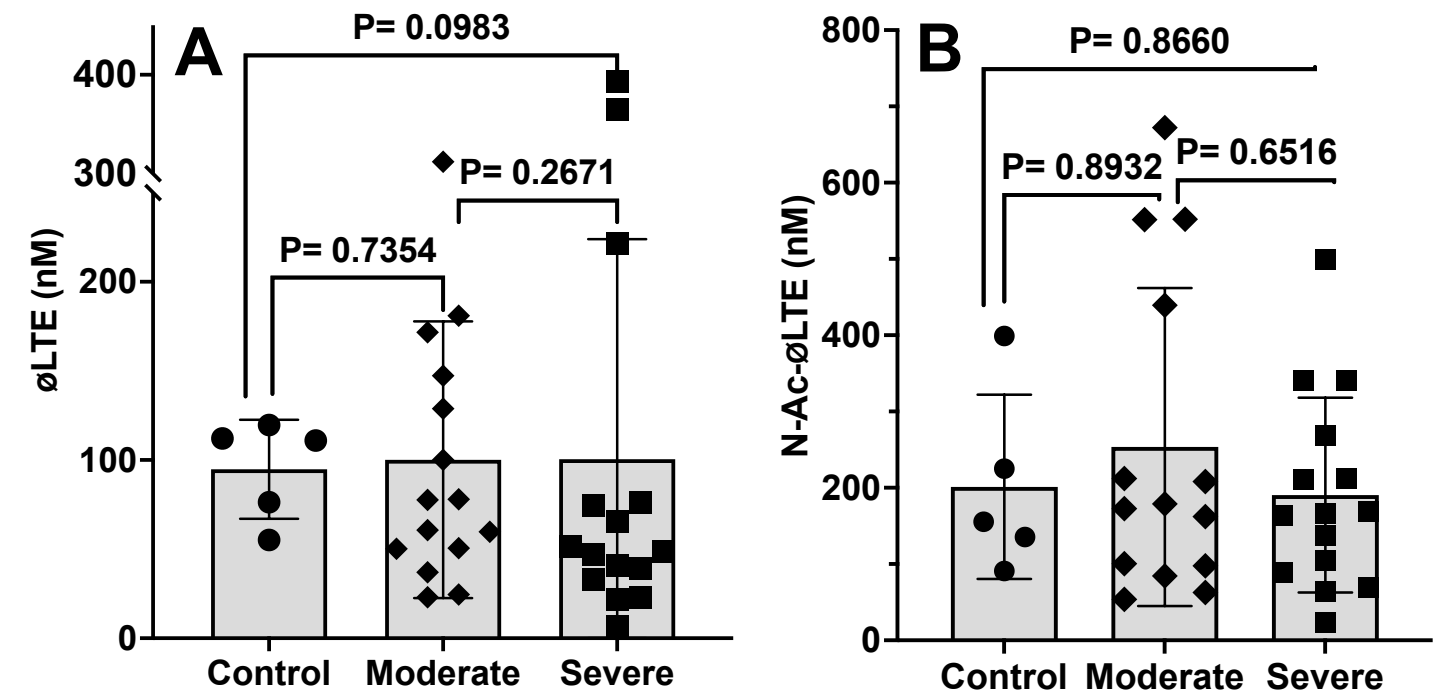

Figure E1. øLTE and N-Ac-øLTE concentrations are abundant in human urine. Each data point is the mean from three independent analyses of each sample and standard deviations are of those means. All the experiments were performed in triplicate. Because Kolmogorov-Smirnov normality test showed non normal distributions (Data S3), significant differences were determined by Mann-Whitney nonparametric analysis. A P value < 0.05 is considered as statistically significant. Control N = 5, Moderate and Severe N = 15. Data are presented as mean ± SD.

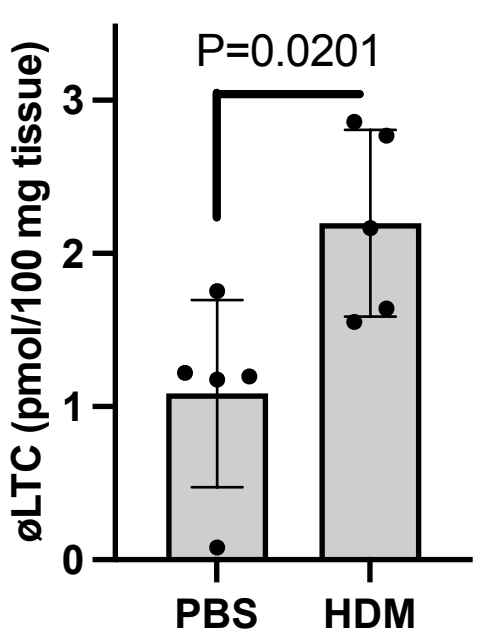

Figure E2. **HDM exposure elevates øLT levels in mouse lung.** Each data point is the mean from three independent analyses of each sample and standard deviations are of those means.

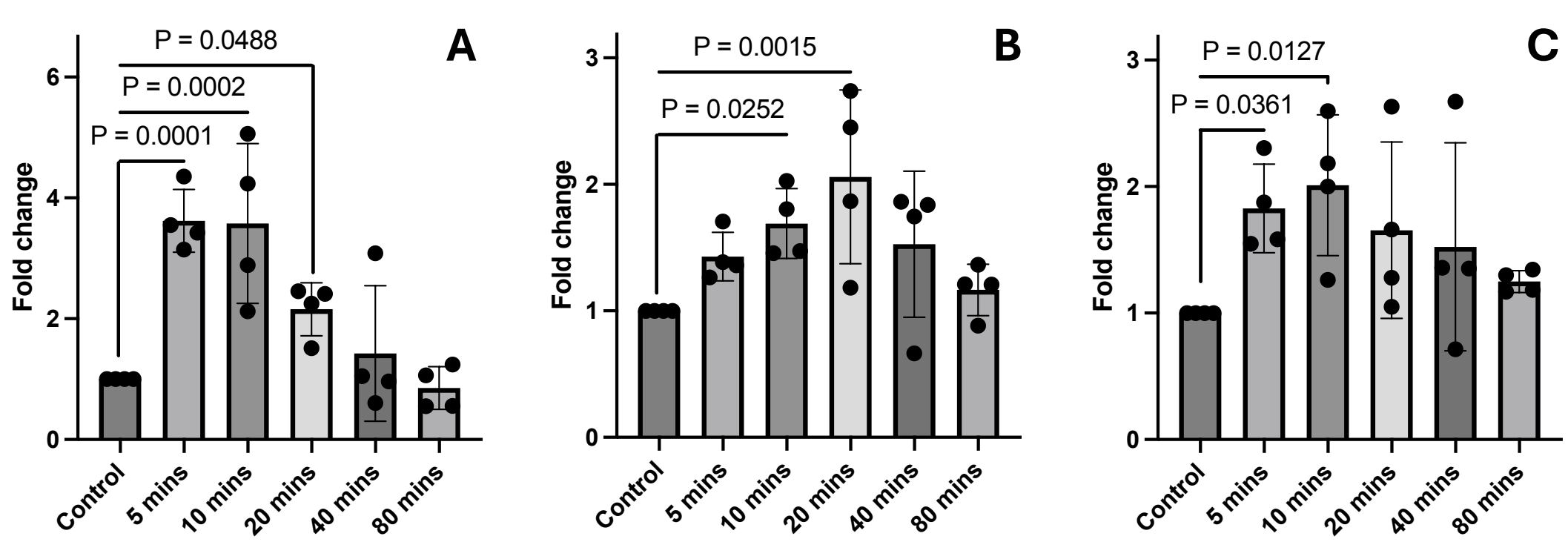

Figure E3. **Phosphorylation of ERK, Akt and NFkB in 16-HBE cells exposed to 200 nM øLTE.** Data are mean ± SD of four independent experiments. For representative immunoblotting images see **Fig. E4**.

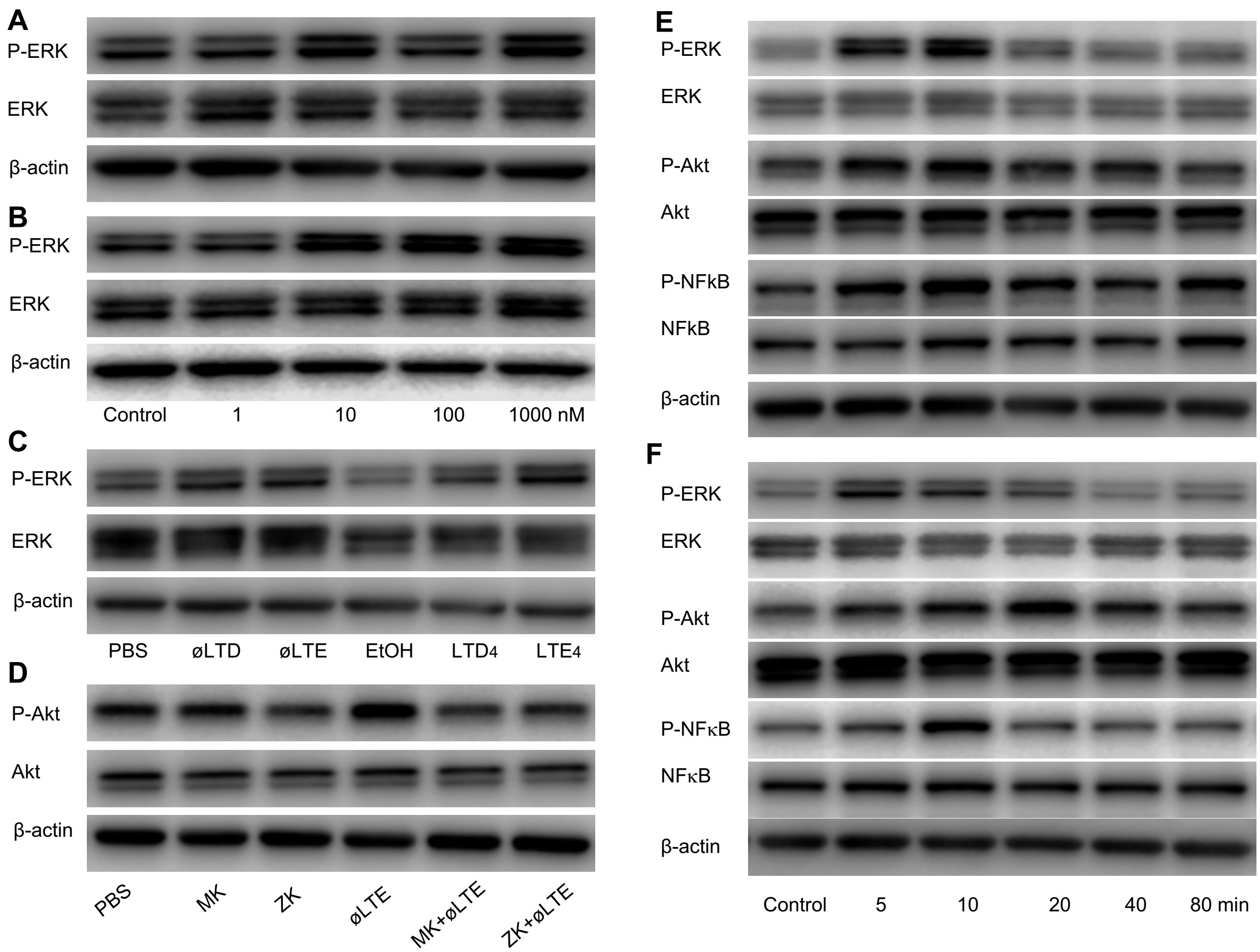

Figure E4. Representative immunoblotting images. A. Phosphorylation of ERK induced by different concentrations of øLTD at 10 minutes.; **B.** Phosphorylation of ERK induced by different concentra-tions of øLTE at 10 minutes.; **C.** Phosphorylation of ERK induced by 200 nM øLTD or øLTE or 100 nM LTD_4 or LTE_4.; **D**. Phosphorylation of Akt induced by 10 min exposure to øLTE (200 nM) with or without inhibition by pretreatment (1 h) with Montelukast (MK, 1 μM) or Zafirlukast (ZK, 1 μM); **E.** øLT-induced phosphorylation of ERK, Akt and NF-kB in 16-HBE cells exposed to 200 nM øLTD; **F.** øLT-induced phosphorylation of ERK, Akt and NF-kB in 16-HBE cells exposed to 200 nM øLTE.

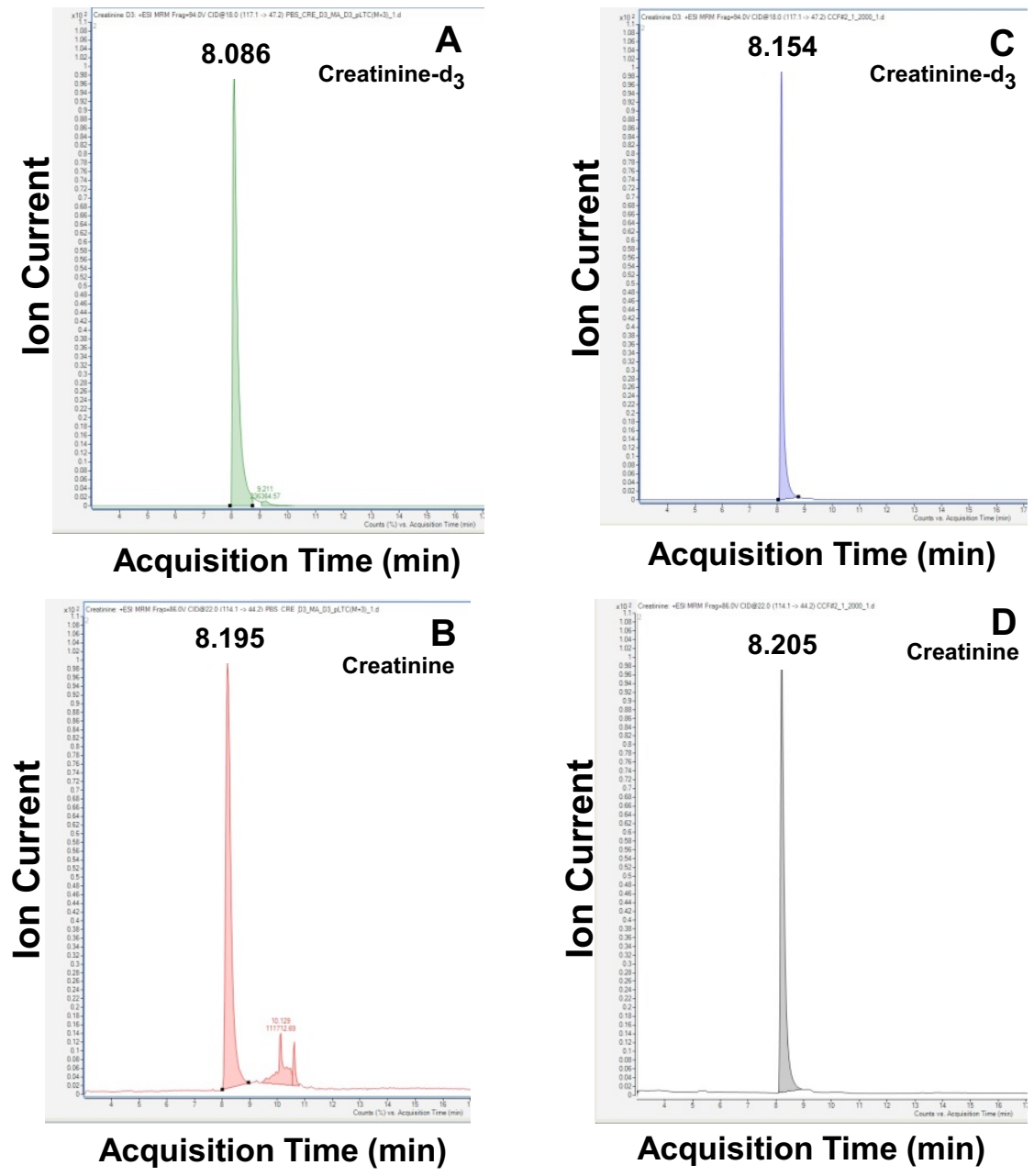

Figure E5A. Representative LC–ESI-MS/MS chromatograms for quantitative analysis of creatinine and the internal standard creatinine-d3: (A) creatinine-d3, 4 pg/injection, (B) creatinine, 4 pg/injection; and these molecules detected in 1:2000 diluted urine sample from a patient with severe asthma (C) creatinine-d3 and (D) creatinine.

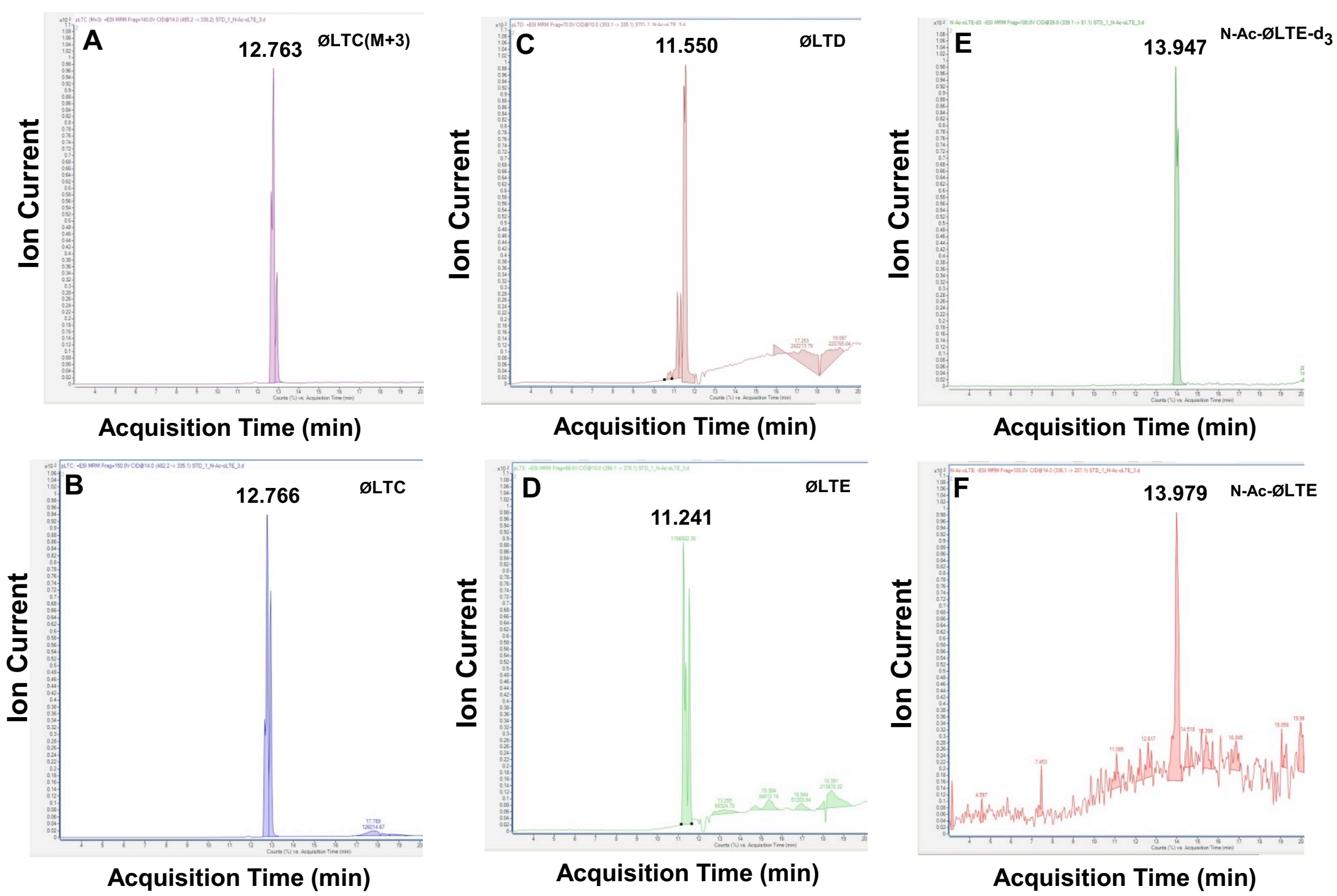

Figure E5B. Representative LC–ESI-MS/MS chromatograms for quantitation of øLTs, N-Ac-øLTE authentic samples (standards) and their respective isotopologues/internal standards (200 pmols each: (A) øLTC(M+3, (B) øLTC, (C) øLTD, (D) øLTE, (E) N-Ac-øLTE-d3 and (F) N-Ac-øLTE.

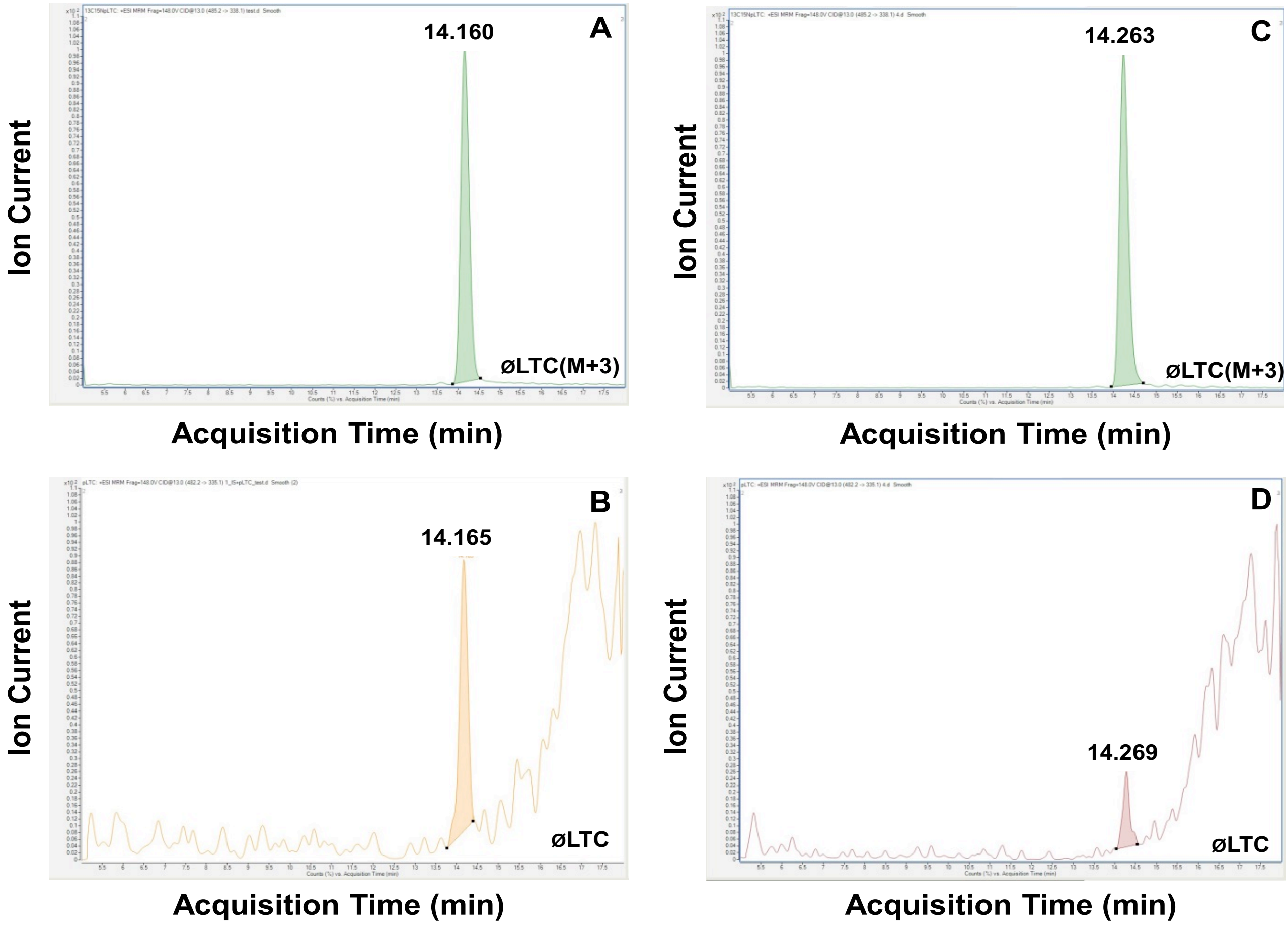

Figure E6. Representative LC–ESI-MS/MS chromatograms for quantitation of øLTC and the internal standard øLTC(M+3): (A) øLTC(M+3), 250 pmols, (B) øLTC, 250 pmols; and these molecules detected in homogenate extracts from HDM-treated mouse lung (C) øLTC(M+3) and (D) øLTC.

Table E1. Antibodies for Western blots.

Antibody	Source	Host
p-p44/42 MAPK (p-ERK1/2)	Cell Signaling Technology, Cat#. 4370S	Rabbit
p44/42 MAPK (ERK1/2)	Cell Signaling Technology, Cat#. 9102S	Rabbit
Phospho-Akt (Ser473) (D9E)	Cell Signaling Technology, Cat#. 4060T	Rabbit
Akt (pan) (C67E7)	Cell Signaling Technology, Cat#. 4691T	Rabbit
Phospho-NF-κB p65	Cell Signaling Technology, Cat#. 3033T	Rabbit
NF-κB p65	Cell Signaling Technology, Cat#. 8242S	Rabbit
β-actin	Cell Signaling Technology, Cat#. 5125S	Rabbit

Table E2. HPLC gradient used for the detection of human urinary øLTs and creatinine.

Time (min)	Solvent A (%)	Solvent B (%)
Initial	95	5
6	98	2
8	85	15
10	65	35
15	5	95
19	5	95
19.1	95	5
30	5	95

Solvent A: 0.1% formic acid in H_2O
Solvent B: 0.1% formic acid in acetonitrile
Column: Hypercarb Porous Graphitic Carbon (2.1x150 mm, 5 µm; 35005-152130) equipped with a guard cartridge (10 mm, 5 mm; 35005-011001)
Flow Rate = 0.3 mL/min

Table E3. MRM transitions, fragmentation energies, collision energies for creatinine, ØLTs and their internal standards

Compound Name	Precursor Ion	Daughter's Ion	Dwelling Time (µsec)	Fraggmentor (V)	Collision Energy (V)	Polarity
Creatinine	114.1	44.2	200	86	22	Positive
Creatinine-d3*	117.1	47.2	200	94	18	Positive
N-Ac-øLTE	336.1	207.1	200	100	14	Positive
N-Ac-øLTE-d3*	339.1	81.1	200	100	26	Negative
øLTC (M+3)*	485.2	338.2	200	140	14	Negative
øLTC	482.2	335.1	200	150	14	Positive
øLTD	353.1	335.1	200	70	10	Positive
øLTE	296.1	278.1	200	98	10	Positive

*- internal standards

Table E4. HPLC gradient used for the detection of murine lung øLTs.

Time (min)	Solvent A (%)	Solvent B (%)
Initial	95	5
6	98	2
8	85	15
10	65	35
15	5	95
19	5	95
19.1	95	5
30	5	95

Solvent A: 0.1% formic acid in H_2O
Solvent B: 0.1% formic acid in acetonitrile
Column: Hypercarb Porous Graphitic Carbon (1.0 x100 mm, 3 mm; 35003-101030) equipped with a guard cartridge (10 mm x 2.1, 3 mm; 35003-012101)
Flow Rate = 0.06 mL/min

Data E1. Levels of øLTs in urine from control volunteers and asthma patients

Data E1A1. Levels of øLTC in urine from control volunteers and asthma patients (per mL/mg Cre)

	øLTC/CRE (pmol/mg_Cre)			øLTC/CRE (pmol/mg_Cre)						
	Asthma Grading				Asthma Grading					
					MEAN				± SD	
Patient (n)	control	moderate	severe	control	moderate	severe		control	moderate	severe
1	1.273041883	4.754768915	32.11205616							
2	1.07232365	3.637313254	20.49447813							
3	1.17173511	2.635089404	11.66223183	1.041733	1.702522	7.1428		0.2088	1.2748	9.126875
4	0.96037045	2.270643808	10.75703413							
5	0.731195351	2.185068157	7.026482015							
6		1.866871828	3.787059068							
7		1.007520909	3.421977042							
8		0.955859429	2.695035456							
9		0.894195668	2.075852288							
10		0.860131613	1.345344759							
11		0.845051544	1.322644742							
12		0.80103025	1.307509654							
13		0.728807338	1.001708828							
14		0.392955382	0.990152053							

Data E1A2. Levels of øLTC in urine from control volunteers and asthma patients (nM)

	øLTC (nM)			øLTC (nM)						
	Asthma Grading				Asthma Grading					
					MEAN				± SD	
Patient (n)	control	moderate	severe	control	moderate	severe		control	moderate	severe
1	2.101451162	26.60085787	62.37394974							
2	1.779347896	4.912761243	39.73385861							
3	2.469646362	9.151535645	12.90930442	2.396061	5.973369	11.944		0.53961	6.378474	16.78278
4	2.401844913	3.704179349	5.261172039							
5	3.22801521	6.080397881	8.26081518							
6		3.751039447	5.171853465							
7		3.698411324	5.701101498							
8		2.452453281	1.034460948							
9		1.716943369	3.82658943							
10		1.99203946	3.989956516							
11		4.016837943	2.723158745							
12		7.015451768	13.32907029							
13		1.074619014	3.917603763							
14		2.177005537	5.85935664							
15		11.25600118	5.070236088							

Data E1B1. Levels of øLTD in urine from control volunteers and asthma patients (per mL/mg Cre)

	øLTD/CRE (pmol/mg_Cre)				øLTD/CRE (pmol/mg_Cre)					
	Asthma Grading					Asthma Grading				
					MEAN				± SD	
Patient (n)	control	moderate	severe	control	moderate	severe		control	moderate	severe
1	20.84086	2.276654	24.12488							
2	10.18615	19.08124	15.2909							
3	4.504513	12.97793	31.86913	8.5418	24.76756	37.1861		7.40997	15.70964	23.134212
4	3.972403	17.30985	36.07912							
5	3.205067	11.19352	35.29691							
6		27.30206	50.53836							
7		38.44226	29.922							
8		46.1798	33.58406							
9		41.00608	83.42138							
10		14.04017	90.92572							
11		16.31941	25.52127							
12		58.31797	14.36687							
13		25.47441	20.20924							
14		16.82451	29.45557							

Data E1B2. Levels of øLTD in urine from control volunteers and asthma patients (nM)

	øLTD (nM)					øLTD (nM)				
	Asthma Grading					Asthma Grading				
					MEAN				± SD	
Patient (n)	control	moderate	severe	control	moderate	severe		control	moderate	severe
1	34.40267	72.84029	46.85979							
2	16.90227	28.46292	56.48085							
3	9.494087	66.26821	108.6353	16.9767	61.31801	70.0736		10.2132	32.70562	38.27611
4	9.934807	10.1104	16.27634							
5	14.14944	104.4651	14.22731							
6		49.68614	76.38186							
7		112.9219	128.9752							
8		109.6086	34.68213							
9		21.79853	118.4472							
10		37.38361	51.63177							
11		70.40567	53.15331							
12		61.32613	120.457							
13		27.59992	56.84349							
14		58.01892	57.04314							
15		88.87387	111.01							

Data E1C1. Levels of øLTE in urine from control volunteers and asthma patients (per mL/mg Cre)

		øLTE/CRE (pmol/mg_Cre)				øLTE/CRE (pmol/mg_Cre)				
		Asthma Grading					Asthma Grading			
					MEAN				± SD	
Patient (n)	control	moderate	severe	control	moderate	severe		control	moderate	severe
1	67.1935	24.96972	113.862							
2	72.0873	49.41515	20.5957							
3	36.2108	64.40789	106.961	44.5853	34.762847	45.6337		23.5275	26.150543	36.78698
4	22.0419	29.98189	14.7587							
5	25.3928	13.46343	54.2728							
6		14.67191	25.876							
7		35.11183	7.62846							
8		107.1919	39.7313							
9		25.24646	52.6358							
10		15.47636	40.7098							
11		20.75269	22.6951							
12		51.79372	16.5167							
13		16.24666	18.2264							
14		17.95022	104.402							

Data E1C2. Levels of øLTE in urine from control volunteers and asthma patients (nM)

		øLTE (nM)					øLTE (nM)			
		Asthma Grading					Asthma Grading			
					MEAN				± SD	
Patient (n)	control	moderate	severe	control	moderate	severe		control	moderate	severe
1	110.918489	147.2814581	221.163148	94.8169	100.149191	100.4777		27.796	77.65687	123.475379
2	119.617272	312.1736003	76.0753987							
3	76.3208844	171.6163766	364.608156							
4	55.1259087	50.17664738	6.65808532							
5	112.101874	180.9410011	21.8760035							
6		59.76186415	39.1081224							
7		60.6833092	32.8815466							
8		100.1127359	41.0303903							
9		50.59845664	74.7358197							
10		23.01619431	23.1169018							
11		77.60760713	47.2672786							
12		77.9858088	48.3850812							
13		24.5122189	65.3494112							
14		37.00237909	51.4464395							
15		128.7682131	393.463774							

Data E1D1. Levels of N-Ac-øLTE in urine from control volunteers and asthma patients (per mL/mg Cre)

	N-Ac-øLTE/CRE (pmol/mg_Cre)				N-Ac-øLTE/CRE (pmol/mg_Cre)					
	Asthma Grading					Asthma Grading				
					MEAN				± SD	
Patient (n)	control	moderate	severe	control	moderate	severe		control	moderate	severe
1	82.0521	72.8995	108.327							
2	93.6494	126.517	37.2786							
3	43.1792	69.10937	62.233	85.8912	86.21765	110.665		46.2294	48.6763	63.1618
4	159.588	35.16391	142.093							
5	50.9878	19.02055	172.423							
6		41.77314	69.2855							
7		62.74964	5.33073							
8		206.8232	259.375							
9		110.1834	118.979							
10		110.0947	156.456							
11		55.39492	78.339							
12		132.4481	85.975							
13		71.17005	120.56							
14		93.69967	132.647							

Data E1D2. Levels of N-Ac-øLTE in urine from control volunteers and asthma patients (nM)

	N-Ac-øLTE (nM)					N-Ac-øLTE (nM)				
	Asthma Grading					Asthma Grading				
					MEAN				± SD	
Patient (n)	control	moderate	severe	control	moderate	severe		control	moderate	severe
1	135.446	551.5561	210.413							
2	155.396	911.3956	137.698							
3	91.0081	439.3872	212.139	201.214	297.3185	190.435		120.735	263.0351	127.729
4	399.121	53.83932	64.1024							
5	225.096	212.2145	69.4996							
6		84.42896	104.716							
7		172.7746	22.9775							
8		178.9151	267.855							
9		97.62801	168.934							
10		100.4498	88.843							
11		552.0797	163.157							
12		208.1666	165.812							
13		62.68322	340.166							
14		162.0924	340.297							
15		672.1667	499.91							

Data E2. Levels of øLTC in the lung homogenates from mice treated with PBS or house dust mite extract in PBS.

	øLTC (pmol/100 mg wet weight)		
Mouse (n)	WT	MEAN	**±SD**
1	1.196	1.086	0.611
2	1.176		
3	0.082		
4	1.755		
5	1.220		
Mouse (n)	HDM	MEAN	**±SD**
1	2.165	2.198	0.610
2	1.641		
3	1.554		
4	2.859		
5	2.770		

Data E3. Kolmogorov-Smirnov Normality Test of the Data

øLTC control (pmol/mg creatinine) distribution determined by the two-sided Kolmogorov-Smirnov statistic (the maximum absolute difference) D(5) = 0.158, Lilliefors p-value > 0.1. Since the p-value is greater than 0.05, there is not sufficient evidence to reject the hypothesis that the data is normal. In other words, it appears that the data points are approximately normally distributed.

øLTC moderate (pmol/mg creatinine) the two-sided Kolmogorov-Smirnov statistic (the maximum absolute difference) D(14) = 0.279, Lilliefors p-value = 0.004. Since the p-value is less than 0.05, the data does not appear to be normally distributed.

øLTC severe (pmol/mg creatinine) the two-sided Kolmogorov-Smirnov statistic (the maximum absolute difference) D(14) = 0.286, Lilliefors p-value = 0.003. Since the p-value is less than 0.05, the data does not appear to be normally distributed.

Q-Q Plots of øLTC (pmol/mg creatinine) Data

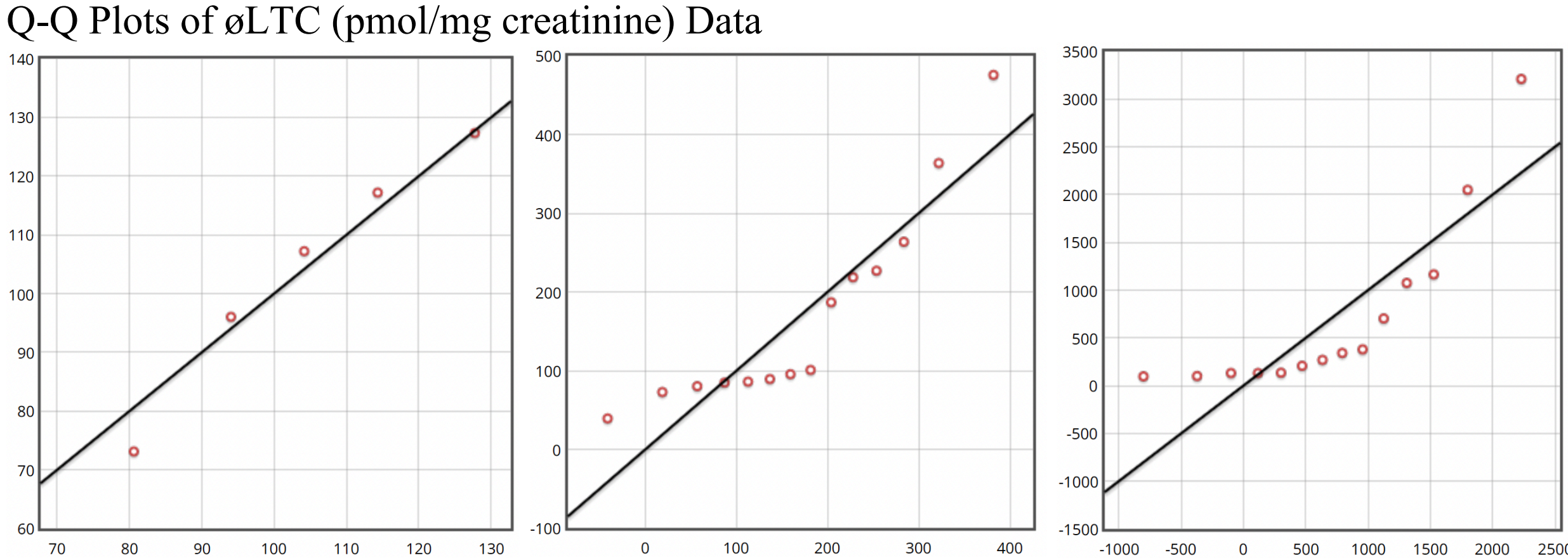

Control Moderate Severe

øLTD control (pmol/mg creatinine) the two-sided Kolmogorov-Smirnov statistic (the maximum absolute difference) D(5) = 0.307, Lilliefors p-value > 0.1. Since the p-value is greater than 0.05, there is not suffcient evidence to reject the hypothesis that the data is normal. In other words, it appears that the data points are approximately normally distributed.
øLTD moderate (pmol/mg creatinine) the two-sided Kolmogorov-Smirnov statistic (the maximum absolute difference) $D(14) = 0.213$, Lilliefors p-value = 0.086. Since the p-value is greater than 0.05, there is not sufficient evidence to reject the hypothesis that the data is normal. In other words, it appears that the data points are approximately normally distributed.
øLTD severe (pmol/mg creatinine) the two-sided Kolmogorov-Smirnov statistic (the maximum absolute difference) D(14) = 0.305, Lilliefors p-value < 0.001. Since the p-value is less than 0.05, the data does not appear to be normally distributed.

Q-Q Plots of øLTD (pmol/mg creatinine) Data

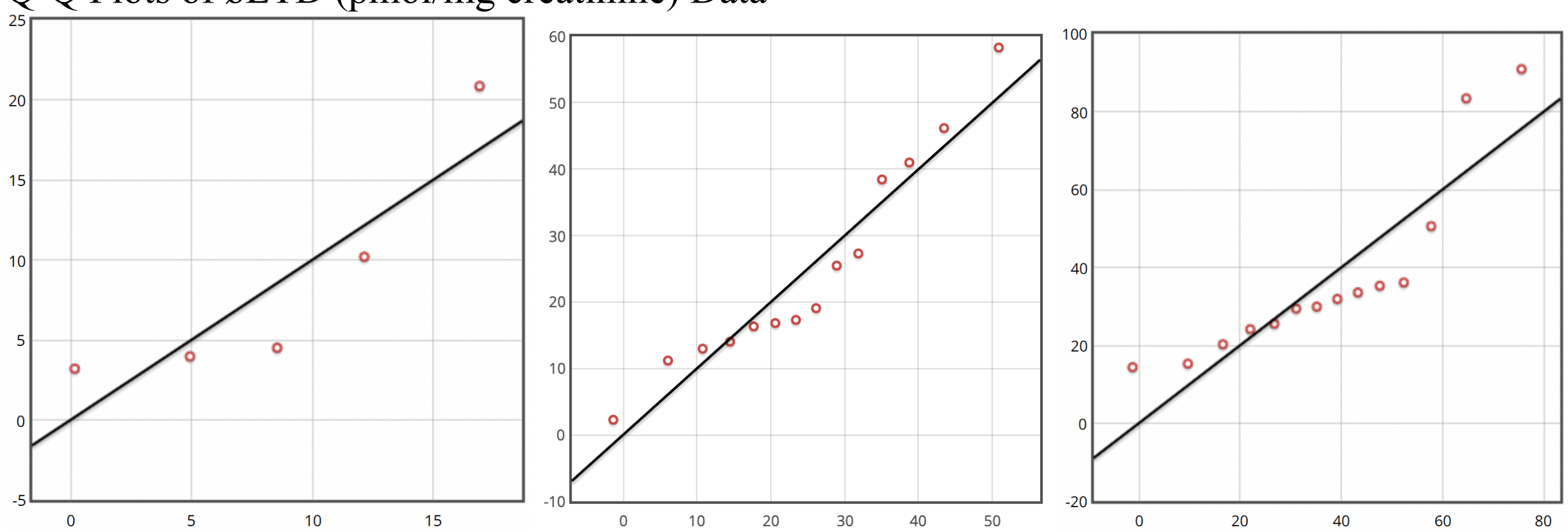

Control Moderate Severe

øLTC control (nM) distribution determined by the two-sided Kolmogorov-Smirnov statistic (the maximum absolute difference) D(5) = 0.246, Lilliefors p-value > 0.1. Since the p-value is greater than 0.05, there is not sufficient evidence to reject the hypothesis that the data is normal. In other words, it appears that the data points are approximately normally distributed.
øLTC moderate (nM) the two-sided Kolmogorov-Smirnov statistic (the maximum absolute difference) D(15) = 0.235, Lilliefors p-value = 0.025. Since the p-value is less than 0.05, the data does not appear to be normally distributed.
øLTC severe (nM) the two-sided Kolmogorov-Smirnov statistic (the maximum absolute difference) D(15) = 0.334, Lilliefors p-value < 0.001. Since the p-value is less than 0.05, the data does not appear to be normally distributed.

Q-Q Plots of øLTC (nM) Data

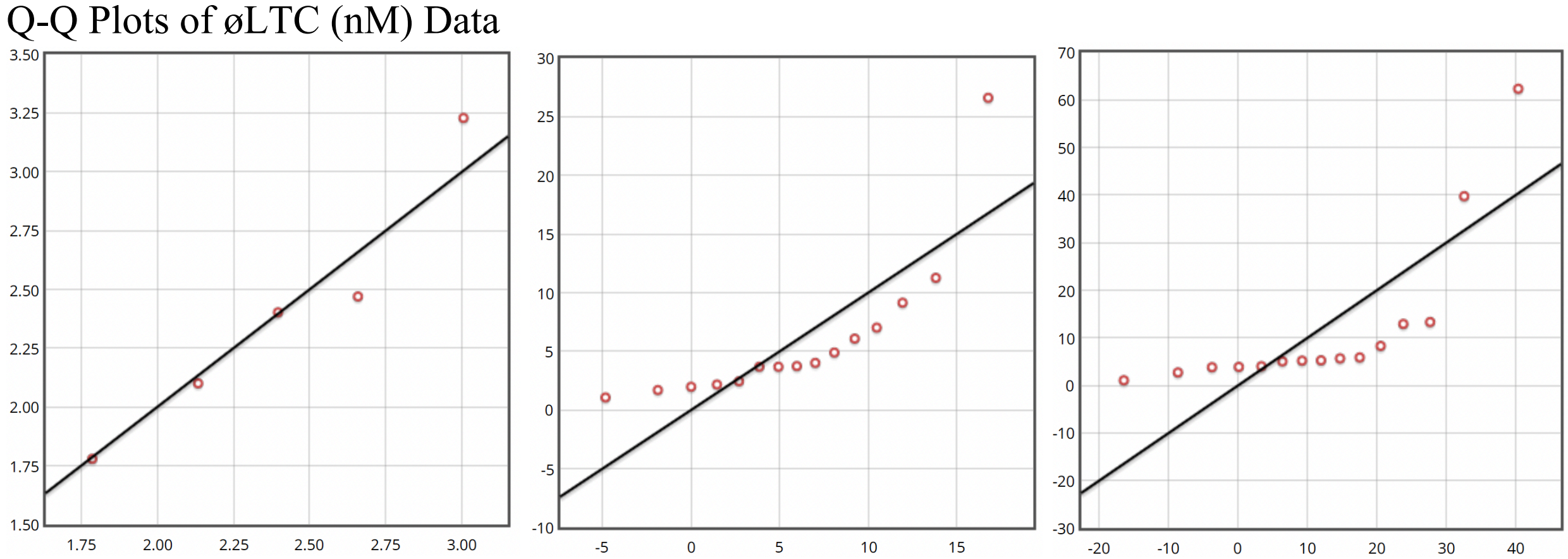

Control Moderate Severe

øLTD control (nM) distribution determined by the two-sided Kolmogorov-Smirnov statistic (the maximum absolute difference) D(5) = 0.303, Lilliefors p-value > 0.1. Since the p-value is greater than 0.05, there is not sufficient evidence to reject the hypothesis that the data is normal. In other words, it appears that the data points are approximately normally distributed.
øLTD moderate (nM) the two-sided Kolmogorov-Smirnov statistic (the maximum absolute difference) D(15) = 0.109, Lilliefors p-value > 0.1. Since the p-value is greater than 0.05, there is not sufficient evidence to reject the hypothesis that the data is normal. In other words, it appears that the data points are approximately normally distributed.
øLTD severe (nM) the two-sided Kolmogorov-Smirnov statistic (the maximum absolute difference) D(15) = 0.233, Lilliefors p-value = 0.027. Since the p-value is less than 0.05, the data does not appear to be normally distributed.

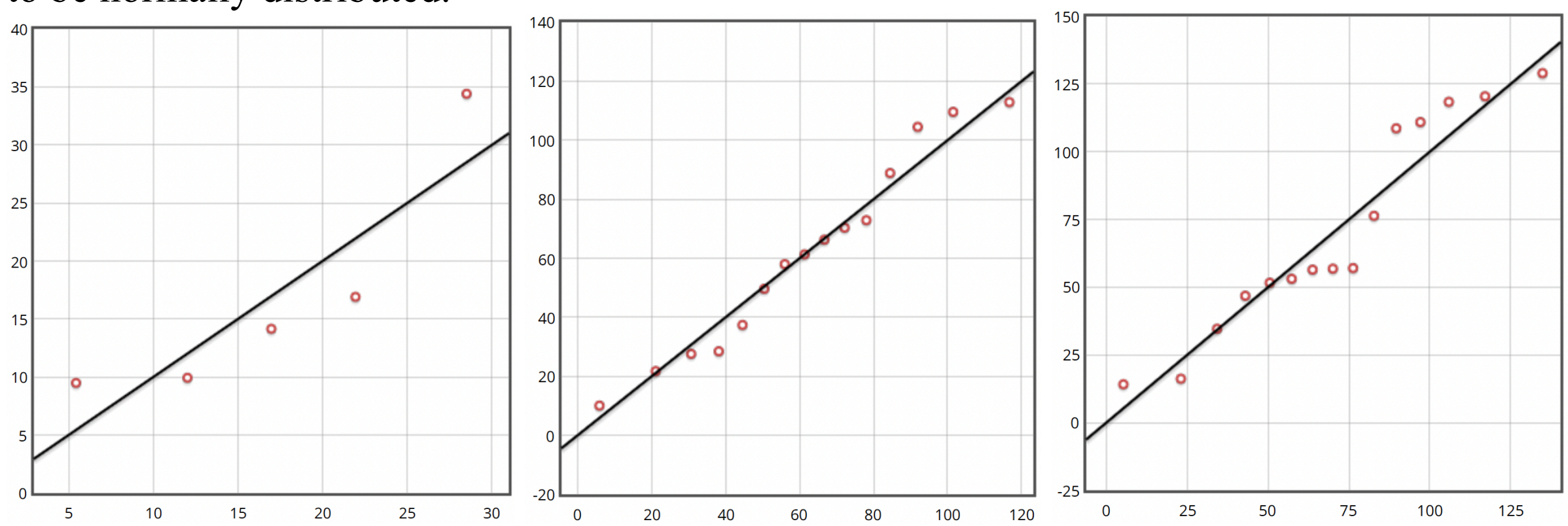

Control Moderate Severe

Data E4. Patient Characteristics.

Column 3	age_Years	gender: male=1 female =2	severity: 0=non severe 1=severe 2=healthy	race: 3= black 5=white 6=other	bmi	FEV1_%	FVC_%	FEV1FVC_%	Max bronchodilator	LT antiagonist in the last 12 months. 0= no, 1=yes	LT antiagonist in the last 3 months, 0= no, 1=yes	FENO ppb	Blood eosinophils, %	øLTC+øLTD (nM)	creatinine (mg/mL)
Urine-1	23	2	0	3	34.2	77.7	105.4	73.3	23.4	0	0	75	0.6	75.6	1.65
Urine-2	52	2	1	3	43.4	102.3	106.4	95.9	7.6	0	0	9	0.7	9.7	1.66
Urine-3	44	2	1	3	29.2	58.6	84.7	69.0	25.9	0	0	26	0.2	26.2	2.11
Urine-4	36	2	0	3	33.2	96.3	94.9	101.1	1.9	1	1	12	0.3	12.3	2.50
Urine-5	48	2	0	5	44.3	48.2	62.7	76.6	11.8	0	0	38	0.3	38.3	4.41
Urine-6*	35	1	1	5	34.1	41.1	78.2	52.3	19.9	0	0	21	0.3	33.4	428
Urine-7	61	2	1	5	27.5	74.0	100.4	73.2	17.9	1	1	19	0.5	21.3	12.50
Urine-8	66	2	1	5	35.3	54.0	84.1	63.9	25.5	0	0	35	0.9	19.5	3.47
Urine-9	27	2	0	3	29.5	88.4	116.0	75.9	18.5	1	1	85	0.5	35.9	0.78
Urine-10	18	2	0	6	19.0	82.2	74.8	109.1	1.6	0	0	94	0.4	85.5	6.04
Urine-11	29	1	2	5	30.5	105.0	100.1	104.4	2.1			26	0.1	94.4	4.44
Urine-12	61	2	2	5	23.7	104.5	120.2	86.3	3.9			11	0.4	26.1	4.14
Urine-13	28	2	2	6	27.2	111.9	124.4	89.5	6.7			19	0.3	11.4	2.85
Urine-14	53	2	2	5	37.8	86.8	93.9	91.7	10.1			18	1.1	19.3	0.47
Urine-15	35	1	2	3	31.2	95.8	94.2	101.7	1.2			33	1	19.1	0.91
Urine-16	31	1	0	3	28.1	94.5	132.0	71.2	10.1	0	0	33	1.8	34	5.01
Urine-17	30	2	0	5	52.4	64.4	83.1	77.2	13.2	0	0	34	0.4	34.8	3.76
Urine-18	38	1	0	5	25.0	73.1	82.4	88.2	5.1	0	0	26	0.6	34.4	0.47
Urine-19	39	1	0	5	26.2	85.9	94.6	90.5	6.8	0	0	13	0.5	26.6	2.28
Urine-20	52	1	1	5	38.0	50.5	62.4	80.6	8.7	1	1	25	0.3	13.5	7.17
Urine-21	54	1	1	3	39.1	34.5	68.5	50.2	11.3	1	1	256	0.3	25.3	1.94
Urine-22	51	2	1	3	29.5	92.9	114.7	80.6	18.0	1	1	18	0.5	256.3	3.69
Urine-23	50	2	1	3	30.2	69.6	70.2	98.6	3.6	1	1	20	0.3	18.5	3.41
Urine-24	49	2	1	5	35.1	76.2	85.4	88.9	8.1	0	0	52	0.2	20.3	0.45
Urine-25	33	2	1	5	44.2	23.2	47.3	48.8	20.5	0	0	12	0.1	52.2	0.40
Urine-26	42	2	0	3	33.0	91.9	91.4	100.1	1.9	1	1	27	0.3	12.1	1.51
Urine-27	31	1	0	3	39.4	85.0	98.9	85.5	5.4	0	0	47	0.6	27.3	4.31
Urine-28	29	2	1	3	60.3	66.0	81.3	80.9	18.7	1	1	35	0.3	47.6	1.03
Urine-29	43	2	1	3	29.6	67.2	81.8	81.8	14.7	0	0	24	0.9	35.3	1.42
Urine-30	53	1	1	5	33.5	70.7	102.8	68.6	25.1	1	1	63	0.6	24.9	0.57
Urine-31	27	2	1	5	45.1	79.2	79.9	98.5	5.6	1	1	22	0.1	63.6	2.08
Urine-32*	21	2	0	3	43.6	66.6	85.2	77.9	17.1	0	0	29	0.3	133.7	0.0013
Urine-33	49	2	0	5	28.4	71.9	97.7	73.2	12.0	1	1	11	0.2	22.1	3.96
Urine-34	25	2	0	5	32.1	83.3	110.1	75.2	15.2	1	0	19	0.3	29.3	2.82
Urine-35	18	1	0	3	18.2	42.7	69.1	61.3	58.1	1	0	61	0.3	11.2	3.77

* These data were excluded from the calculation of urinary øLT levels (nM/mg cretinine) because the measured creatinine levels are outliers.

Correlation Between FEV1/FVC (%) versus Asthma Severity.

Analysis of lung function as FEV1/FVC (%) for patients in our control and severe asthma cohorts revealed the expected impaired lung function in the patients with severe asthma (**Fig. E7**).

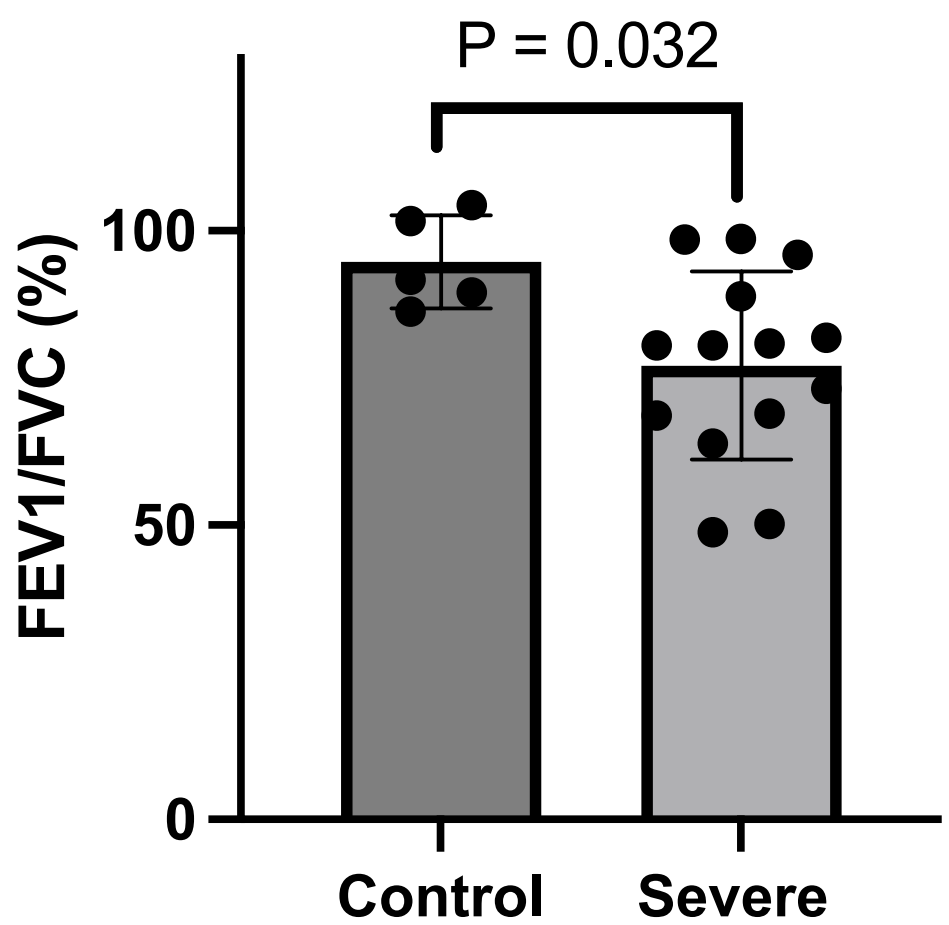

Fig. E7. Lung function is impaired in patients with severe asthma. Significant differences were determined by Mann-Whitney nonparametric analysis of patient data (Data S4). A P value < 0.05 is considered as statistically significant. Control N = 5, Severe N = 14. Data are presented as mean ± SD.

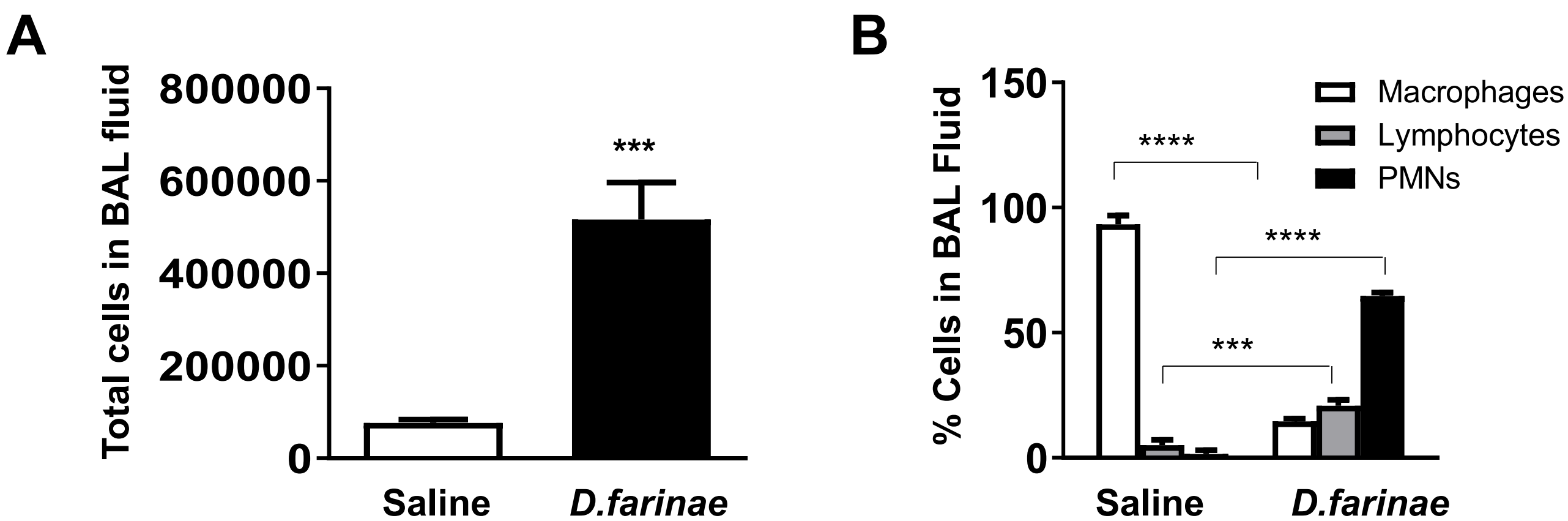

Figure E8. WT mice treated with *D. farinae* exhibit enhanced inflammation. C57BL/6 mice were treated with saline or *D. farinae* intranasally. **A.** total cell count and **B.** differential cell counts from the BAL fluid were determined. Data are reported as mean ± SD ($n = 5$). Statistical significance of pairwise comparisons calculated by two-way ANOVA. ***$p < .001$, and ****$p < .0001$.
